# Mutational analysis of *dishevelled* genes in zebrafish reveals distinct functions in embryonic patterning and gastrulation cell movements

**DOI:** 10.1371/journal.pgen.1007551

**Published:** 2018-08-06

**Authors:** Yan-Yi Xing, Xiao-Ning Cheng, Yu-Long Li, Chong Zhang, Audrey Saquet, Yuan-Yuan Liu, Ming Shao, De-Li Shi

**Affiliations:** 1 School of Life Sciences, Shandong University, Jinan, China; 2 Affiliated Hospital of Guangdong Medical University, Zhanjiang, China; 3 Sorbonne Universités, UPMC Univ Paris, CNRS UMR7622, IBPS-Develompental Biology Laboratory, Paris, France; University of Pennsylvania School of Medicine, UNITED STATES

## Abstract

Wnt signaling plays critical roles in dorsoventral fate specification and anteroposterior patterning, as well as in morphogenetic cell movements. Dishevelled proteins, or Dvls, mediate the activation of Wnt/ß-catenin and Wnt/planar cell polarity pathways. There are at least three highly conserved Dvl proteins in vertebrates, but the implication of each Dvl in key early developmental processes remains poorly understood. In this study, we use genome-editing approach to generate different combinations of maternal and zygotic *dvl* mutants in zebrafish, and examine their functions during early development. Maternal transcripts for *dvl2* and *dvl3a* are most abundantly expressed, whereas the transcript levels of other *dvl* genes are negligible. Phenotypic and molecular analyses show that early dorsal fate specification is not affected in maternal and zygotic *dvl2* and *dvl3a* double mutants, suggesting that the two proteins may be dispensable for the activation of maternal Wnt/ß-catenin signaling. Interestingly, convergence and extension movements and anteroposterior patterning require both maternal and the zygotic functions of Dvl2 and Dvl3a, but these processes are more sensitive to Dvl2 dosage. Zygotic *dvl2* and *dvl3a* double mutants display mild axis extension defect with correct anteroposterior patterning. However, maternal and zygotic double mutants exhibit most strongly impaired convergence and extension movements, severe trunk and posterior deficiencies, and frequent occurrence of cyclopia and craniofacial defects. Our results suggest that Dvl2 and Dvl3a products are required for the activation of zygotic Wnt/ß-catenin signaling and Wnt/planar cell polarity pathway, and regulate zygotic developmental processes in a dosage-dependent manner. This work provides insight into the mechanisms of Dvl-mediated Wnt signaling pathways during early vertebrate development.

## Author summary

The embryogenesis of most animals is first supported by maternal gene products accumulated in the oocyte, and then by the expression of genes from the zygote. In all vertebrates, there are at least three Dishevelled (Dvl) proteins, which play critical roles in normal development and human diseases. They are both maternally and zygotically expressed, and can activate the ß-catenin-dependent Wnt pathway that regulates gene expression and cell fate, and the ß-catenin-independent Wnt pathway that orchestrates cell movements. In zebrafish embryo, Dvl2 and Dvl3a are most abundant, but their functions are not fully understood. We find that maternally and zygotically expressed Dvl2 plays a predominant role in the elongation of the anteroposterior axis, and the expression of genes involved in the development of the posterior region. Dvl3a cooperates with Dvl2 in these processes. Analyses after loss-of-function of these genes indicate that deficiency of maternal and zygotic Dvl2 and Dvl3a results in embryos with cyclopia, craniofacial defects, and severe abnormality in the trunk and posterior regions. Many human birth defects and other diseases, like cancer, are attributed to the dysfunction of the Wnt pathways. Our results help to understand the mechanisms of Dvl-mediated Wnt pathway activation, and the causes of developmental disorders.

## Introduction

The specification of the dorsoventral (DV) axis is tightly linked to, and ultimately determines, the processes that establish the anteroposterior (AP) pattern in vertebrate embryos. A large body of work has elucidated the induction and patterning processes underlying DV and AP polarities [1, 2]. It is well established that, in *Xenopus* and zebrafish, maternal canonical Wnt/ß-catenin signaling is activated on the future dorsal side following fertilization, and ß-catenin is absolutely required for the establishment of the primary DV asymmetry through cooperation with other factors, such as members of the Nodal family [1–5]. During gastrulation, dorsolateral cells converge toward the dorsal midline, while dorsal midline cells undergo extension along the AP axis. These movements, called convergence and extension (CE), not only provide the driving force for gastrulation, but also make an important contribution to the elongation of AP axis. The conserved non-canonical Wnt/PCP (planar cell polarity) signaling plays a key role in CE movements in all vertebrates [6–13]. Thus, disruption of Wnt/ß-catenin and Wnt/PCP signaling pathways can result in severe defects in the formation of embryonic axes.

Dishevelled (Dvl) is a key intracellular signaling molecule that mediates the activation of both Wnt/ß-catenin and Wnt/PCP pathways during early development [6, 8, 14–18]. Functional analyses in *Xenopus* suggest that Dvl2 (also called Xdsh) exhibits dorsalizing and neuralizing activity [14], and controls polarized cell behaviors that are required for CE movements during gastrulation [15]. However, its requirement for the activation of maternal Wnt/ß-catenin signaling in dorsal axis specification has not been clearly established, and remains largely enigmatic [17]. Simultaneous depletion of maternally expressed *dvl2* and *dvl3* from *Xenopus* oocytes using morpholino antisense oligonucleotides has no obvious effect on the expression of maternal Wnt/ß-catenin target genes and the specification of dorsal axis [19]. This negative result may be due to the presence of stored punctae of Dvl proteins in the oocyte cortex, which translocate to the dorsal region soon after fertilization [20], or due to the insufficiency of maternal *dvl* mRNA depletion. Therefore, appropriate genetic approaches will be necessary to determine whether Dvls function upstream of ß-catenin in early dorsal fate specification.

There are three *dvl* genes (*dvl1*, *dvl2* and *dvl3*) in human, mouse, and *Xenopus*, and at least five in zebrafish [21, 22]. They are highly conserved and broadly expressed throughout early development [21, 23]. Extensive analyses of mutant phenotypes in mice have uncovered both unique and redundant functions among the three *Dvl* genes [24–27]. Although single mutants for these mouse *Dvl* genes generally survived to adulthood, abnormal neural tube closure and defective organogenesis were observed in different combinations of double mutants, which also cause embryonic lethality [25–27]. This, combined with the development *in utero*, make it less convenient to assay Dvl implication in early axis patterning and morphogenetic processes. In *Xenopus*, triple knockdown of *dvl1*, *dvl2* and *dvl3* led to CE defects [21], which were similar as those resulted from overexpression of Xdd1, a Dvl2 (Xdsh) mutant lacking the PDZ domain [28]. Interestingly, Dvl1 and Dvl2 were found to be involved in neural crest specification and somite segmentation, while Dvl3 was required to maintain muscle gene expression [21]. These observations reveal a distinct requirement of Dvl proteins for Wnt signaling in regulating the expression of developmental genes. However, at present, the relative contribution of individual Dvl protein in CE movements that are dependent on the Wnt/PCP pathway has not been clearly determined, making this important question quite open for further investigation.

One of the critical functions of Wnt signaling during early development is the requirement of zygotic Wnt/ß-catenin pathway for ventroposterior development in *Xenopus* and zebrafish embryos. In contrast to maternal Wnt/ß-catenin signaling that specifies dorsal fate, zygotic Wnt/ß-catenin signaling inhibits anterior development by activating the expression of different target genes that specify ventral and posterior tissues [1–5]. How different *dvl* genes are implicated in this process also remains poorly understood. Another difficulty in studying Dvl function is the presence of abundant maternal *dvl* transcripts, as shown both in *Xenopus* [14, 19], and in zebrafish [29, 30]. These maternal products play an important role to support early developmental processes, and when translated into proteins and stored during oogenesis, could not be targeted by knockdown approaches. Also, zygotic homozygous mutants often do not survive to fertile adulthood to produce maternal and zygotic (MZ) mutant embryos for the analysis of maternal gene function. Taken together, it is clear that the maternal and zygotic contributions of *dvl* genes in different patterning and morphogenetic processes remain elusive, and merits further investigation.

In the present study, we take advantage of the genome-editing approach to generate different combinations of maternal and zygotic *dvl* mutants in zebrafish. Transcriptomic analysis revealed that, among the five *dvl* genes, only *dvl2* and *dvl3a* are maternally and abundantly expressed, whereas the transcript levels of the other three *dvl* genes (*dvl1a*, *dvl1b*, *dvl3b*) are negligible [29]. By creating targeted mutations, we find that MZ*dvl2* mutants display most severe CE and craniofacial defects, but with correct AP patterning. In *dvl2* and *dvl3a* double mutants, *dvl3a* dosage exerts a permissive effect on the loss of *dvl2* in CE movements and posterior development. By further targeting the wild-type (WT) allele in triallelic mutant embryos to generate mosaic germline transmissible double homozygous adults, we obtained MZ*dvl2*;MZ*dvl3a* offspring. These mutants show correct dorsal fate specification, but display severe CE defects and develop trunk and posterior deficiencies, as well as cyclopia. These observations indicate that Dvl2 and Dvl3a may be not required for maternal Wnt/ß-catenin pathway activation. Instead, Dvl2 plays a major role in Wnt/PCP and zygotic Wnt/ß-catenin signaling. Our findings thus help to better understand the function of Dvl proteins in different patterning and morphogenetic processes.

## Results

### MZ*dvl2* mutants display axis extension and craniofacial defects

**Fig 1:**
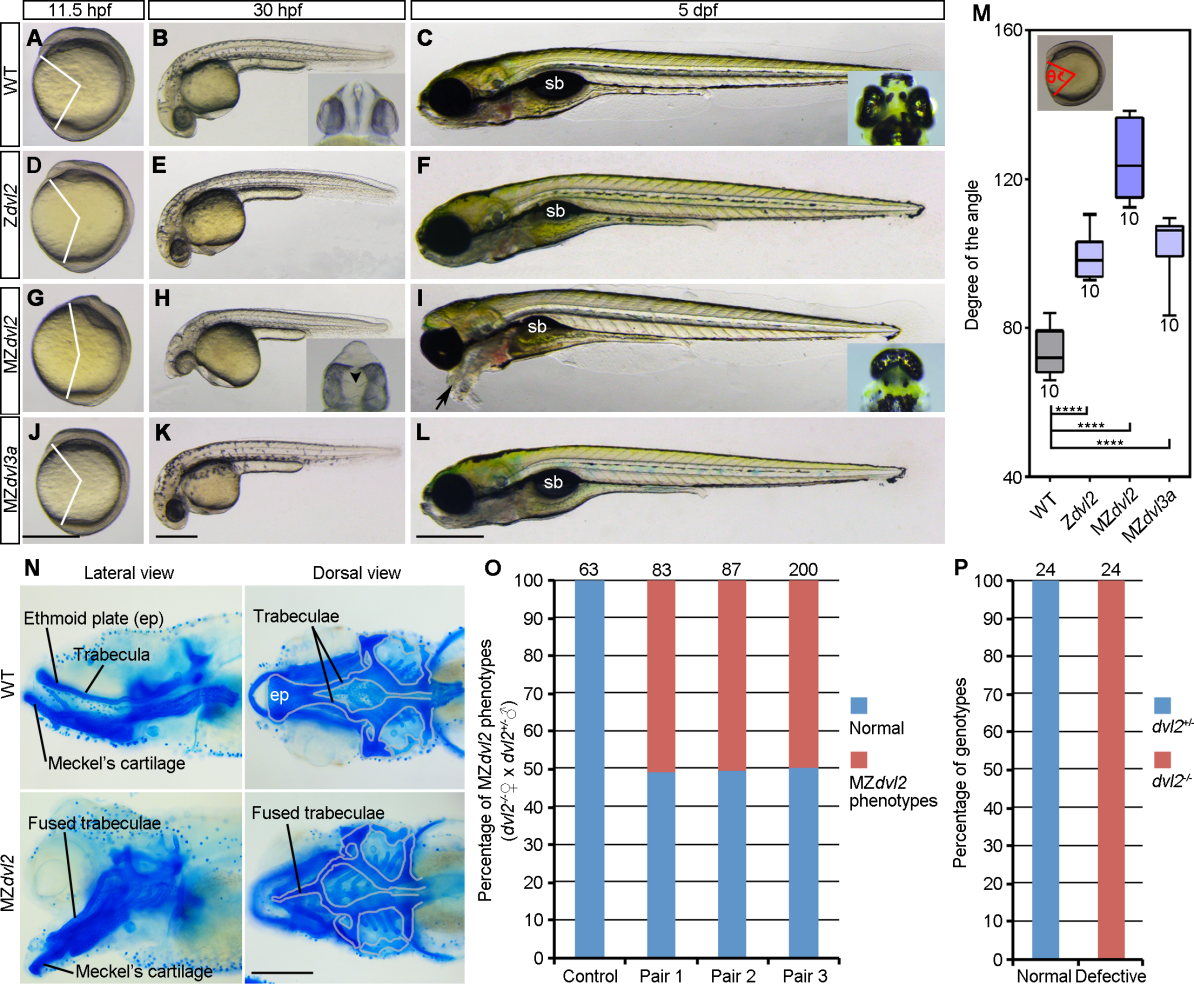
Analysis of *dvl2* and *dvl3a* mutant phenotypes. (A-C) WT embryos, the insets show the eyes in ventral view at 30 hpf and in dorsal view at 5 dpf. (D-F) Z*dvl2* mutants, obtained by crosses between heterozygous *dvl2*^+/-^ carriers, display weak axis extension defect at 11.5 hpf. They are normal at 30 hpf, and show reduced swim bladder (sb) at 5 hpf. (G-I) MZ*dvl2* mutants, obtained from crosses between female *dvl2*^-/-^ fish and male *dvl2*^+/-^ fish, display a reduced AP axis at 11.5 hpf and 30 hpf. They present fused eyes at 30 hpf (inset, ventral view; arrowhead indicates fused lenses), and develop craniofacial defects and cyclopia (inset, dorsal view), with pharyngeal cartilages protruding outward (arrow) at 5 dpf. (J-L) MZ*dvl3a* mutants from crosses between *dvl3a*^-/-^ carriers display weak axis extension defect, but are indistinguishable from WT embryos at 30 hpf and 5 dpf. (M) Statistical analysis of the extent of axis extension delay. The embryos were imaged at 11.5 hpf followed by genotyping. Those embryos with expected genotypes were used to measure the angle between the anterior end and posterior end, with vertex at the geometric center of the embryo (inset). Bars represent the mean ± s.d. from indicated numbers of embryos (****, *P*<0.0001). (N) Alcian blue staining of head cartilages at 5 dpf. Cartilage structures of the basicranium are outlined in grey, showing the fusion of trabeculae and the absence of ethmoid plate (ep) in MZ*dvl2* mutants. (O) Quantitative analysis of MZ*dvl2* mutant phenotypes at 5 dpf in offspring from three independent female *dvl2*^-/-^ and male *dvl2*^+/-^ fish pairs. Control embryos were obtained from crosses between female WT fish and male *dvl2*^+/-^ fish. Numbers on the top of each column indicate total embryos analyzed. (P) Genotyping of *dvl2* mutants with normal and defective phenotypes. All embryos with a normal phenotype are *dvl2*^+/-^ mutants, whereas all defective embryos are MZ*dvl2* mutants. Numbers on the top of each column indicate total embryos genotyped from three independent fish pairs. Scale bars: (A, D, G, J) 400 μm; (B, E, H, K) 400 μm; (C, F, I, L) 400 μm; (N) 100 μm.

We used transcription activator-like effector nucleases (TALENs) genome-editing approach to generate mutant lines for the five zebrafish *dvl* genes (*dvl1a*, *dvl1b*, *dvl2*, *dvl3a*, *dvl3b*). All indel mutations led to premature stop codons in the transcripts, and resulted in proteins truncated either at the DIX or the PDZ domain (S1–S5 Figs). Analysis of the phenotypes at different stages indicated that, except for *dvl2*, maternal and zygotic mutants for the other *dvl* genes developed normally, and could survive to adulthood and were fertile (Fig 1 and S6 Fig). Because *dvl2* and *dvl3a* represent the most abundantly expressed maternal transcripts (S7 Fig), we focused our analyses on these two mutant lines. Compared with WT embryos at different stages (Fig 1A–1C), Z*dvl2* mutants showed weakly reduced AP axis extension at 11.5 hpf (hours post-fertilization), as judged by the degree of the angle between the anterior end and posterior end, with vertex at the geometric center of the embryo (Fig 1M). This defect was largely recovered at 30 hpf (Fig 1E). At 5 dpf (days post-fertilization), Z*dvl2* mutants displayed essentially a normal AP axis, except for the presence of a smaller gas-filled swim bladder (Fig 1F). However, only about half of these Z*dvl2* mutants could survive to adulthood, and only about two-third of the survived adult female fish could spawn, whereas all male Z*dvl2* mutants were not fertile due to the absence of courtship behavior.

To obtain MZ*dvl2* embryos, we crossed female *dvl2*^-/-^ fish with male *dvl2*^+/-^ fish. At 11.5 hpf, about half of the resulting embryos showed more severe axis extension defect (Fig 1G and 1M). At 30 hpf, they exhibited a shortened AP axis, associated with a reduced yolk extension, which are characteristics of defective CE movements (Fig 1H). At this stage, MZ*dvl2* mutants also displayed an obvious cyclopic phenotype (compare insets in Fig 1B and 1H). At 5 dpf, although MZ*dvl2* mutants had a similar length of AP axis as WT embryos, abnormalities in the head region were clearly apparent. These include severe craniofacial defects, and cyclopia or fused eyes (compare insets in Fig 1C and 1I). In particular, pharyngeal arches were not correctly positioned, and eventually protruded outward (Fig 1I). Alcian blue staining of larval head cartilages indicated that, among other abnormalities, the pair of trabeculae was fused and the ethmoid plate was absent (Fig 1N). All these phenotypes are reminiscent of impaired Wnt/PCP signaling and defective extension of axial tissues, which are frequently observed in other Wnt/PCP-specific mutants, such as *trilobite*/*vangl2*, and *slb*/*wnt11* [31–33]. Most strikingly, the protrusion outward of pharyngeal cartilages is much similar as the “bulldog” facial phenotype described in *slb*/*wnt11* mutant [32–34]. A more detailed analysis of these late phenotypes is beyond the scope of this study, but it will be interesting for future work. Due to these defects, MZ*dvl2* mutant embryos could not survive beyond 5 dpf. Genotyping by allele-specific PCR (S1 Table and S8 Fig) of large numbers of severely affected embryos derived from three independent fish pairs confirmed that they were indeed MZ*dvl2* mutants (Fig 1O and 1P). In contrast to MZ*dvl2* mutants, the late phenotype of MZ*dvl3a* mutants was indistinguishable from that of WT embryos (Fig 1J–1L), although statistical analysis revealed a weakly reduced AP axis extension at 11.5 hpf (Fig 1M). These results show that both maternal and zygotic Dvl2 make an important contribution to CE movements. They also suggest that deficiency of maternal and zygotic Dvl2 or Dvl3a is not sufficient to affect DV and AP patterning.

By RT-PCR analysis, we found that maternal *dvl2* mutant transcripts were subjected to nonsense-mediated mRNA decay (NMD) at cleavage stages, which was further confirmed by in situ hybridization. Maternal *dvl3a* mutant transcripts also underwent NMD, but to a lesser extent. We then checked whether there was a mutual compensation between *dvl2* and *dvl3a*. No significant change in the level of maternal *dvl2* transcripts was found in MZ*dvl3a* mutants, whereas the level of maternal *dvl3a* transcripts showed a weak decrease in M*dvl2* mutants (S9 Fig). We also attempted to verify if Dvl2 or Dvl3a protein was absent in the mutants by western blotting using available antibodies, but failed to detect any specific signal either in WT or in mutant embryos. Alternatively, to examine whether *dvl2* and *dvl3a* mutant transcripts may be translated by using an alternative ATG or by a stop codon bypass mechanism, we cloned WT and mutant *dvl2* and *dvl3a* coding sequences upstream of myc sequences, and injected the corresponding mRNA (100 pg) into zebrafish embryos. Analysis by western blotting did not detect any signal in embryos injected with these mutant mRNAs (S10A and S10B Fig). This suggests that *dvl2* and *dvl3a* mutant transcripts should not be translated into proteins.

### Zygotic Dvl2 and Dvl3a cooperate to regulate axis extension

**Fig 2:**
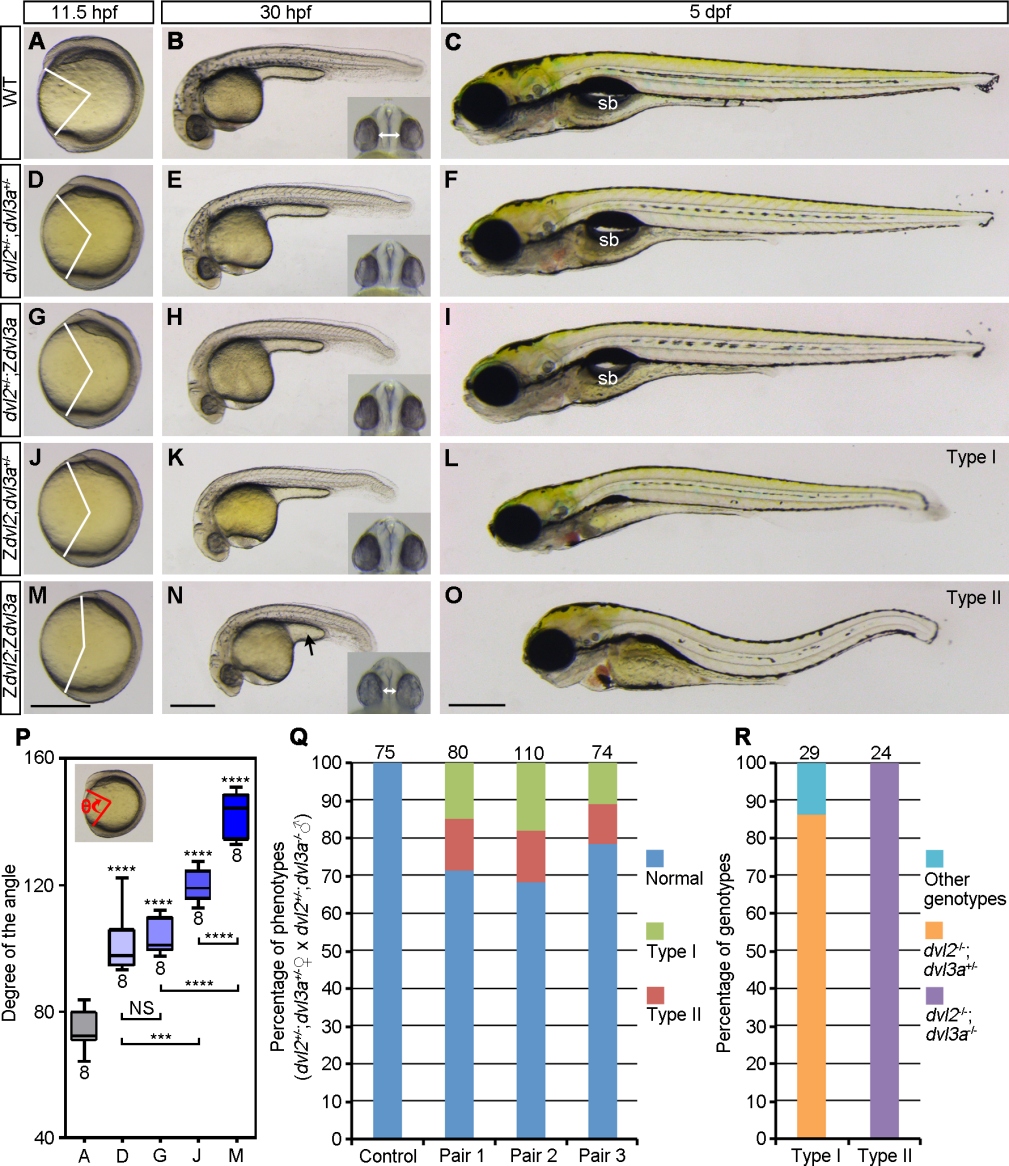
Zygotic Dvl2 and Dvl3a in axis extension. Mutant phenotypes were compared with WT embryos at indicated stages. Insets show eye phenotypes in ventral view, with bidirectional arrows indicating the distance of the two eyes. (A-C) WT embryos. (D-F) Double heterozygous *dvl2*^+/-^;*dvl3a*^+/-^ mutants, from crosses between female *dvl2*^-/-^ fish and male *dvl3a*^-/-^ fish, show weakly delayed axis extension at 11.5 hpf, but are phenotypically normal at later stages. (G-I) Triallelic *dvl2*^+/-^;Z*dvl3a* mutants,from crosses between *dvl2*^+/-^;*dvl3a*^+/-^ carriers, display weak axis extension defect at 11.5 hpf and 30 hpf, and recover at 5 dpf. (J-L) Triallelic Z*dvl2*;*dvl3a*^+/-^ mutants, from crosses between *dvl2*^+/-^;*dvl3a*^+/-^ carriers, exhibit more obvious axis extension defect at 11.5 hpf and 30 hpf, and develop shortened AP axis, compressed head, and reduced swim bladder at 5 dpf, which are referred as type I phenotype. (M-O) Z*dvl2*;Z*dvl3a* mutants, from crosses between female *dvl2*^+/-^;*dvl3a*^+/-^ fish and male *dvl2*^+/-^;Z*dvl3a* fish, show more strong axis extension defect at 11.5 hpf and 30 hpf, and present a severe type II phenotype, with shortened and wavy axis, craniofacial defects, and complete disappearance of swim bladder (sb) at 5 dpf. (P) Statistical analysis of the extent of axis extension delay, after genotyping of imaged embryos at 11.5 hpf. Capital letters of the abscissa correspond to the images at 11.5 hpf. Bars represent the mean ± s.d. from indicated numbers of embryos, and asterisks above the bars refer to comparison with WT embryos (***, *P*<0.001; ****, *P*<0.0001; NS, not significant). (Q) Quantitative analysis of type I and type II phenotypes at 5 dpf from three independent female *dvl2*^+/-^;*dvl3a*^+/-^ and male *dvl2*^+/-^;*dvl3a*^-/-^ fish pairs. Control embryos were from crosses between female WT fish and male *dvl2*^+/-^;*dvl3a*^-/-^ fish. (R) Genotypes of type I and type II embryos. Numbers on the top of each column indicate total embryos analyzed. Scale bars: (A, D, G, J, M) 400 μm; (B, E, H, K, N) 400 μm; (C, F, I, L, O) 400 μm.

To analyze the function of Dvl2 and Dvl3a dosages in AP axis extension and patterning, we first generated double heterozygous *dvl2*^+/-^;*dvl3a*^+/-^ mutants by crosses between female Z*dvl2* and male Z*dvl3a*. Compared to WT embryos (Fig 2A–2C), *dvl2*^+/-^;*dvl3a*^+/-^ mutants showed weak, but significant delay in axis extension at 11.5 hpf (Fig 2D and 2P). They were completely normal at 30 hpf and 5 dpf (Fig 2E and 2F), and developed to fertile adults, indicating that Wnt/ß-catenin signaling was not affected, and Wnt/PCP signaling was weakly affected during gastrulation. We then intercrossed *dvl2*^+/-^;*dvl3a*^+/-^ fish to analyze the phenotypes of triallelic mutants. Similar as *dvl2*^+/-^;*dvl3a*^+/-^ mutants, *dvl2*^+/-^;Z*dvl3a* mutants displayed weak axis extension defect at 11.5 hpf (Fig 2G and 2P), and slightly shortened AP axis at 30 hpf (Fig 2H). These mutants also recovered to a nearly normal phenotype at 5 dpf (Fig 2I). However, the extent of axis extension delay was more pronounced in Z*dvl2*;*dvl3a*^+/-^ mutant embryos at 11.5 hpf (Fig 2J and 2P), which further developed a shortened AP axis at 30 hpf (Fig 2K), and a compressed head at 5 dpf (Fig 2L). These embryos, which were grouped as type I, presented a more severely affected phenotype than Z*dvl2* mutants and did not survive to adulthood. This suggests that removal of one *dvl3a* allele could enhance CE defects in Z*dvl2* mutants.

Since *dvl2*^+/-^;Z*dvl3a* mutants could survive to adulthood and were fertile, the male fish could be used for generating Z*dvl2*;Z*dvl3a* double mutants, after crosses with female *dvl2*^+/-^;*dvl3a*^+/-^ fish. We found that double Z*dvl2*;Z*dvl3a* mutants displayed more strongly affected axis extension at 11.5 hpf (Fig 2M and 2P), and developed a shortened AP axis, with a reduced yolk extension at 30 hpf (Fig 2N). The distance between the two eyes was also reduced (compare insets in Fig 2B and 2N). All these phenotypes are suggestive of impaired CE movements. These embryos, grouped as type II, further developed bent axis and craniofacial defects at 5 dpf (Fig 2O). From three independent fish pairs, we found that the occurrence of the defective axis extension phenotype in the resulting offspring was quite reproducible because similar proportions of type I (13%) and type II (15%) mutant embryos have been obtained (Fig 2Q and 2R), which could be expected from the crosses between *dvl2*^+/-^;*dvl3a*^+/-^ and *dvl2*^+/-^;Z*dvl3a* fish. Since both head and tail were present in zygotic double homozygous mutants, this indicates that half of the maternal Dvl2 and Dvl3a products should be largely sufficient to support AP patterning.

### Maternal contribution of Dvl2 and Dvl3a in axis extension and AP patterning

**Fig 3:**
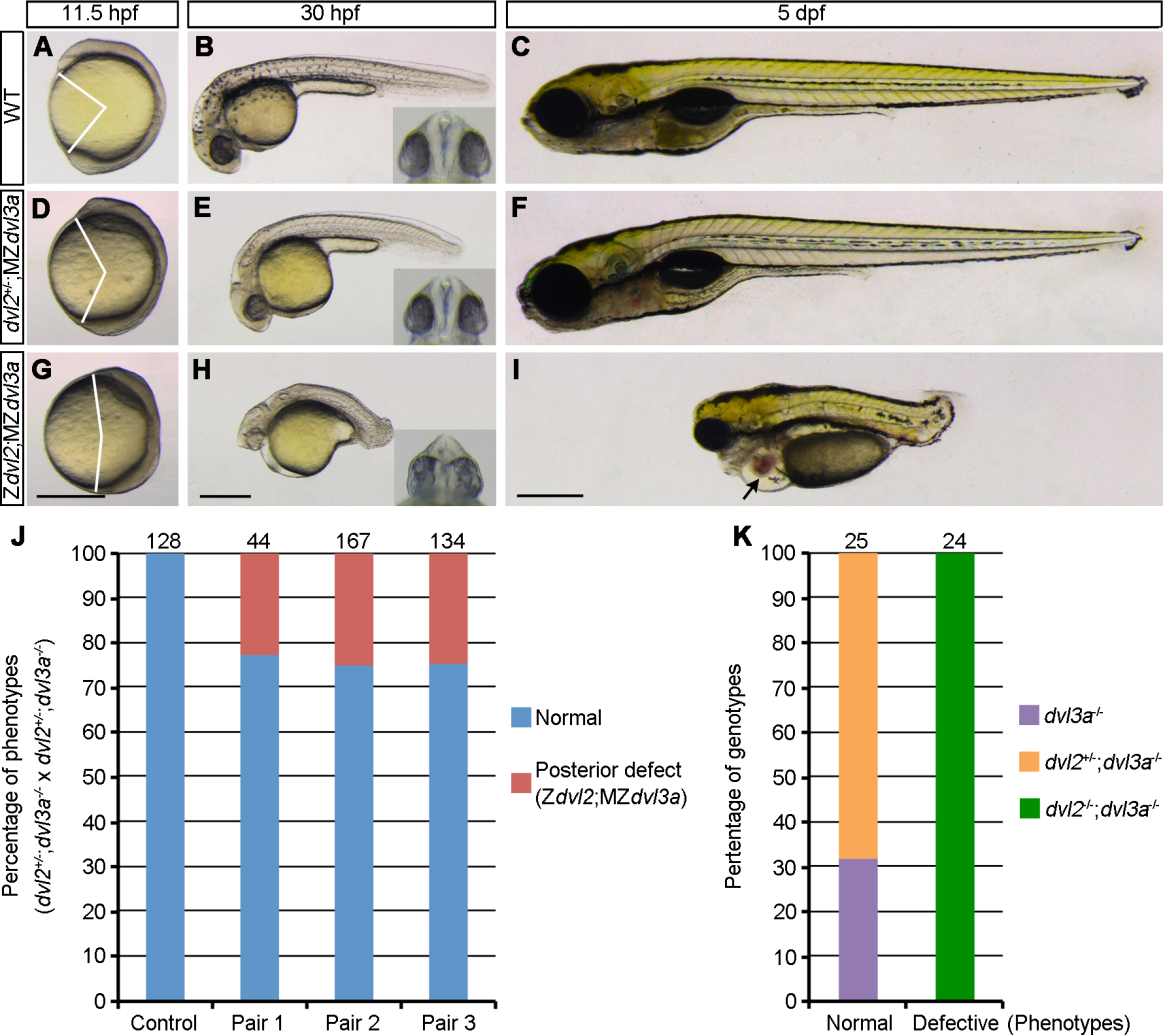
Dvl2 and Dvl3a dosages in axis extension and AP patterning. Both *dvl2*^+/-^;MZ*dvl3a* and Z*dvl2;*MZ*dvl3a* mutants were from crosses between *dvl2*^+/-^;*dvl3a*^-/-^ carriers. Representative mutant embryos were imaged at indicated stages. Eye phenotypes are shown in the insets as ventral view. (A-C) WT embryos. (D-F) *dvl2*^+/-^;MZ*dvl3a* mutants display moderate axis extension defect at 11.5 hpf and 30 hpf, and recover to a normal phenotype at 5 dpf. (G-I) Z*dvl2;*MZ*dvl3a* mutants show strong axis extension defect at different stages, and display caudal truncation, craniofacial defects, cardiac edema (arrow), and fused eyes or cyclopia (inset) at 30 hpf and at 5 dpf. (J) Quantitative analysis of the posterior truncation phenotype at 5 dpf in offspring derived from three independent *dvl2*^+/-^;*dvl3a*^-/-^ carriers. Control embryos were from crosses between female WT fish and male *dvl2*^+/-^;*dvl3a*^-/-^ fish. Posterior deficiency is present in the offspring from all three fish pairs with a proportion that follows the Mandel inheritance (about 25%). Numbers on the top of each column indicate total embryos analyzed. (K) Genotyping of normal and posteriorly truncated embryos. All defective embryos are *dvl2*^-/-^;*dvl3a*^-/-^ mutants. Scale bars: (A, D, G) 400 μm; (B, E, H) 400 μm; (C, F, I) 400 μm.

Since AP patterning does not seem to be affected in Z*dvl2*;Z*dvl3a* mutants, we examined the maternal contribution of Dvl2 and Dvl3a in this process. Intercrosses between *dvl2*^+/-^;Z*dvl3a* triallelic fish could generate three types of mutant offspring, including MZ*dvl3a*, *dvl2*^+/-^;MZ*dvl3a*, and Z*dvl2*;MZ*dvl3a*. Analysis of the phenotypes followed by genotyping indicated that, compared to WT embryos (Fig 3A–3C), *dvl2*^+/-^;MZ*dvl3a* mutants displayed mild axis extension defect at 11.5 hpf (Fig 3D). At 30 hpf, these embryos were short in length, but AP patterning was not affected because head and tail regions were correctly formed (Fig 3E). At 5 dpf, they completely recovered to a normal phenotype (Fig 3F), and eventually developed to fertile adult. This indicates that, in the absence of Dvl3a, maternal and zygotic Dvl2 product derived from one allele is sufficient for AP patterning, although Wnt/PCP pathway activation is reduced at early stages.

Strikingly, Z*dvl2;*MZ*dvl3a* mutants displayed strong axis extension defect at 11.5 hpf (Fig 3G), indicating severely impaired Wnt/PCP signaling. At 30 hpf and 5 dpf, axis extension and AP patterning defects became particularly prominent. These mutants displayed a reduced head size with cyclopia or fused eyes (Fig 3H and 3I). This phenotype is likely caused by perturbed midline development, and is a fish-specific consequence independent of the affected genetic pathway [35]. Cardiac edema was also evident at 5 dpf (Fig 3I). Importantly, Z*dvl2;*MZ*dvl3a* mutants exhibited a severely reduced body length, with posterior truncation (Fig 3H and 3I). We then intercrossed three independent *dvl2*^+/-^;Z*dvl3a* fish pairs, and found that the appearance of severe axis extension defect associated with posterior deficiency was quite reproducible in the resulting offspring, with an average of 24.6% that corresponds to the Mendel inheritance (Fig 3J). Genotyping of these severely affected embryos confirmed that they were indeed Z*dvl2;*MZ*dvl3a* mutants (Fig 3K). This analysis indicates that, in the MZ*dvl3a* background, the reduction of maternal Dvl2 dosage along with the absence of zygotic Dvl2 products strongly impairs Wnt/PCP-dependent axis extension, and leads to defective posterior patterning.

### Generation of MZ*dvl2*;MZ*dvl3a* double mutants

**Fig 4:**
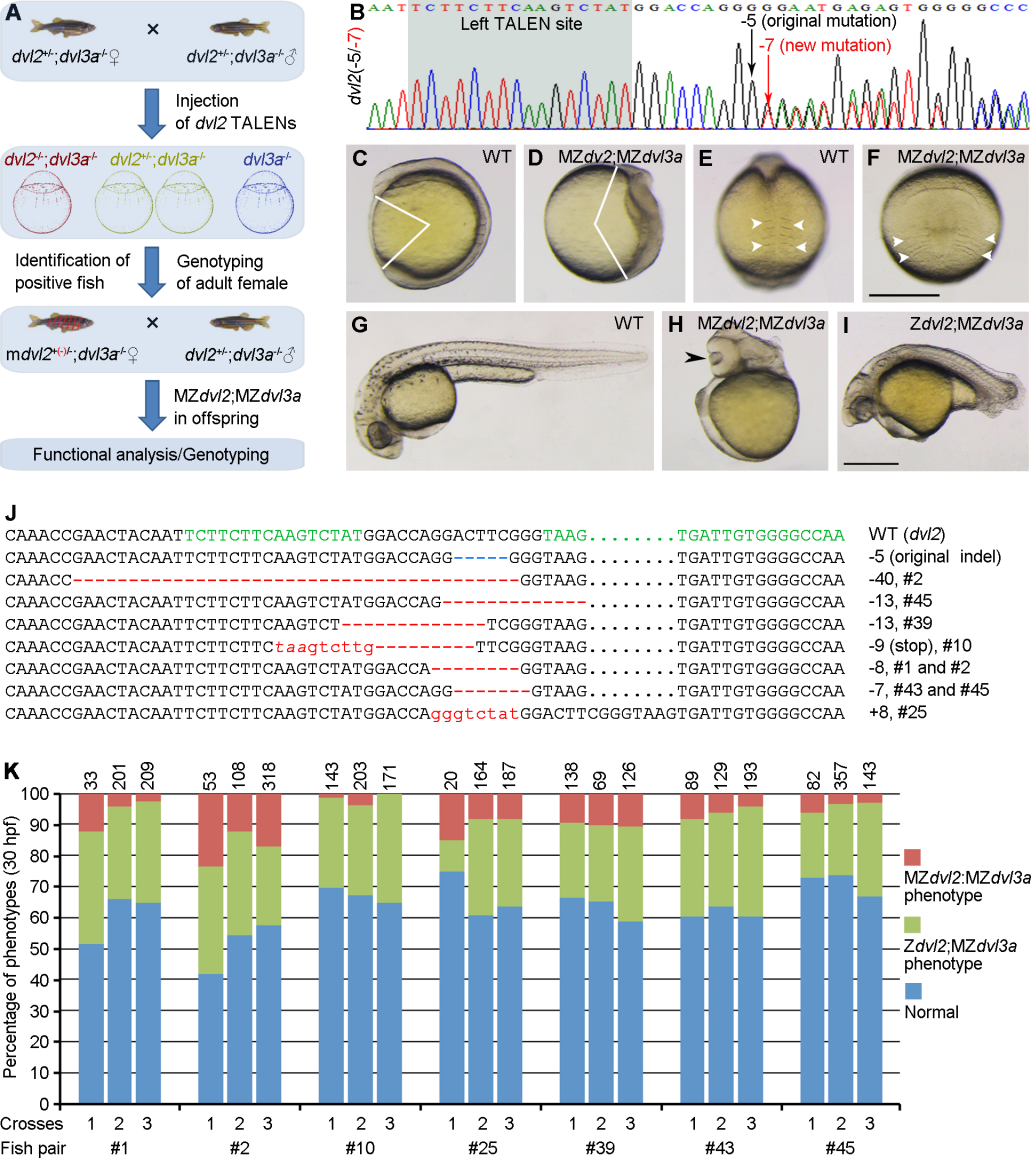
Analysis of MZ*dvl2*;MZ*dvl3a* mutants. (A) Schema illustrating the strategy to generate female mosaic m*dvl2*^+(-)/-^;*dvl3a*^-/-^ adult fish with a new mutant allele (red) in *dvl2*. MZ*dvl2*;MZ*dvl3a* mutant embryos are present at varied proportions in the offspring from crosses between female m*dvl2*^+(*-*)/-^;*dvl3a*^-/-^ and male *dvl2*^+/-^;*dvl3a*^-/-^ fish, depending on the efficiency of germline mutations in the remaining *dvl2* WT allele. (B) An example of the sequencing chromatogram with both the original mutant allele and a new indel in *dvl2* locus. (C-F) Lateral (C, D) and dorsal (E, F) views of a representative WT embryo (C, E), and an MZ*dvl2*;MZ*dvl3a* mutant (D, F) at 11.5 hpf. Notice that the mutant embryo displays most severely impaired AP axis and convergence of paraxial mesoderm, with strongly widened somites (arrowheads). (G) A WT embryo at 30 hpf. (H) Lateral view of a representative MZ*dvl2*;MZ*dvl3a* mutant at 30 hpf shows deficiency of trunk and posterior regions, and cyclopia (arrowhead; see also S12 Fig). (I) The phenotype of a Z*dvl2*;MZ*dvl3a* mutant with characteristic caudal truncation at 30 hpf. (J) Genotyping of *dvl2* alleles in MZ*dvl2*;MZ*dvl3a* mutant embryos from 7 independent fish pairs. A novel indel (red) along with the original mutation (blue) are present in the mutants. The left and right TALEN targeting sites are indicated in green. Dots are introduced to optimize sequence alignment. (K) Quantitative analyses of the occurrence of MZ*dvl2*;MZ*dvl3a* and Z*dvl2*;MZ*dvl3a* mutants among offspring from 7 independent fish pairs. Each fish pair was crossed three times, and numbers on the top of each column indicate total embryos scored. Scale bar: (C-F) 400 μm; (G-I) 400 μm.

Z*dvl2;*Z*dvl3a* double mutants could not survive to adulthood, this prevented us from obtaining maternal and zygotic double mutants to fully address the maternal contribution of Dvl2 and Dvl3a in DV and AP patterning. To circumvent this obstacle, we used a strategy to generate mosaic *dvl2*^*-/-*^;*dvl3a*^*-/-*^ fertile adult fish, by disrupting the remaining *dvl2* WT allele in *dvl2*^+/-^;*dvl3a*^*-/-*^ embryos derived from intercrosses between triallelic *dvl2*^+/-^;*dvl3a*^*-/-*^ fish (Fig 4A). To obtain viable mosaic adult fish, low amounts of the original *dvl2* TALENs mRNAs (100 pg each) were injected in these triallelic mutant embryos at 1-cell stage. The resulting adult female fish were crossed with male *dvl2*^+/-^;*dvl3a*^*-/-*^ fish, and genotyping of the offspring by allelic-specific PCR followed by sequencing was performed to screen female fish carrying a new germline transmissible *dvl2* mutant allele (Fig 4B). Those positive female fish were designated as m*dvl2*^+(*-*)/-^;*dvl3a*^*-/-*^, for mosaic *dvl2* homozygous genotype. By this approach, if mutations of the remaining *dvl2* WT allele occur in some germ cells, MZ*dvl2*;MZ*dvl3a* offspring could be obtained through crosses between female m*dvl2*^+(*-*)/-^;*dvl3a*^*-/-*^ fish and male *dvl2*^+/-^;*dvl3a*^*-/-*^ fish (S11 Fig). Indeed, this strategy has allowed us to generate rather efficiently m*dvl2*^+(*-*)/-^;*dvl3a*^*-/-*^ fish. Among a total of 45 female adults tested, 7 female fish produced offspring with a varied proportion of extremely severe phenotype. At 11.5 hpf, these embryos displayed most strongly reduced axis extension associated with sharply widened paraxial mesoderm (Fig 4C–4F). At 30 hpf, they exhibited severe trunk and posterior deficiencies, but the head region was still present (Fig 4G and 4H). At 5 dpf, all these embryos developed cyclopia or fused eyes (S12 Fig). Other siblings either presented posterior truncation, which were genotyped as Z*dvl2*;MZ*dvl3a* mutants (Fig 4I), or developed relatively normally, which were genotyped as *dvl2*^+/-^;MZ*dvl3a* triallelic mutants or MZ*dvl3a* mutants. These embryos could be expected from the crosses, since a high proportion of germ cells with one *dvl2* WT allele should be still present in m*dvl2*^+(*-*)/-^;*dvl3a*^*-/-*^ fish.

We then genotyped all the most severely affected embryos to confirm the presence of separate mutations in the *dvl2* alleles. As expected, analysis of the sequencing chromatograms revealed that, in addition to the original mutated allele that has a deletion of 5 nucleotides, different new indels were also detected in these embryos (Fig 4J). It is unlikely that the re-injected *dvl2* TALENs could target the original mutant allele in *dvl2*^+/-^;*dvl3a*^*-/-*^ embryos, since we verified that they had no effect in *dvl2*^-/-^ embryos, and it has been shown the TALEN pair had no gene modification activity when separated by 11 nucleotides or less [36], which is the case for the original *dvl2* mutant allele. Thus, we can conclude that the extremely severe defects were specific to MZ*dvl2*;MZ*dvl3a* mutants, and were caused by the deficiency of maternal and zygotic Dvl2 and Dvl3a products. When the phenotypes of offspring from three independent crosses between a fixed pair of female m*dvl2*^+(*-*)/-^;*dvl3a*^-/-^ and male *dvl2*^+/-^;*dvl3a*^-/-^ fish were analyzed, we reproducibly obtained most severely affected embryos, although the proportion varied among fish pairs, or between crosses from a fixed fish pair (Fig 4K). Some female m*dvl2*^+(*-*)/-^;*dvl3a*^-/-^ fish (#2, #25, #39) produced a relatively high proportion of MZ*dvl2*;MZ*dvl3a* mutant embryos, ranging from 10% to 24%, depending on the crosses. Thus, by generating mosaic double mutants, we revealed a maternal requirement for Dvl2 and Dvl3a in AP patterning, and in axis extension.

### Dorsal fate specification is not affected in MZ*dvl2*;MZ*dvl3a* mutants

**Fig 5:**
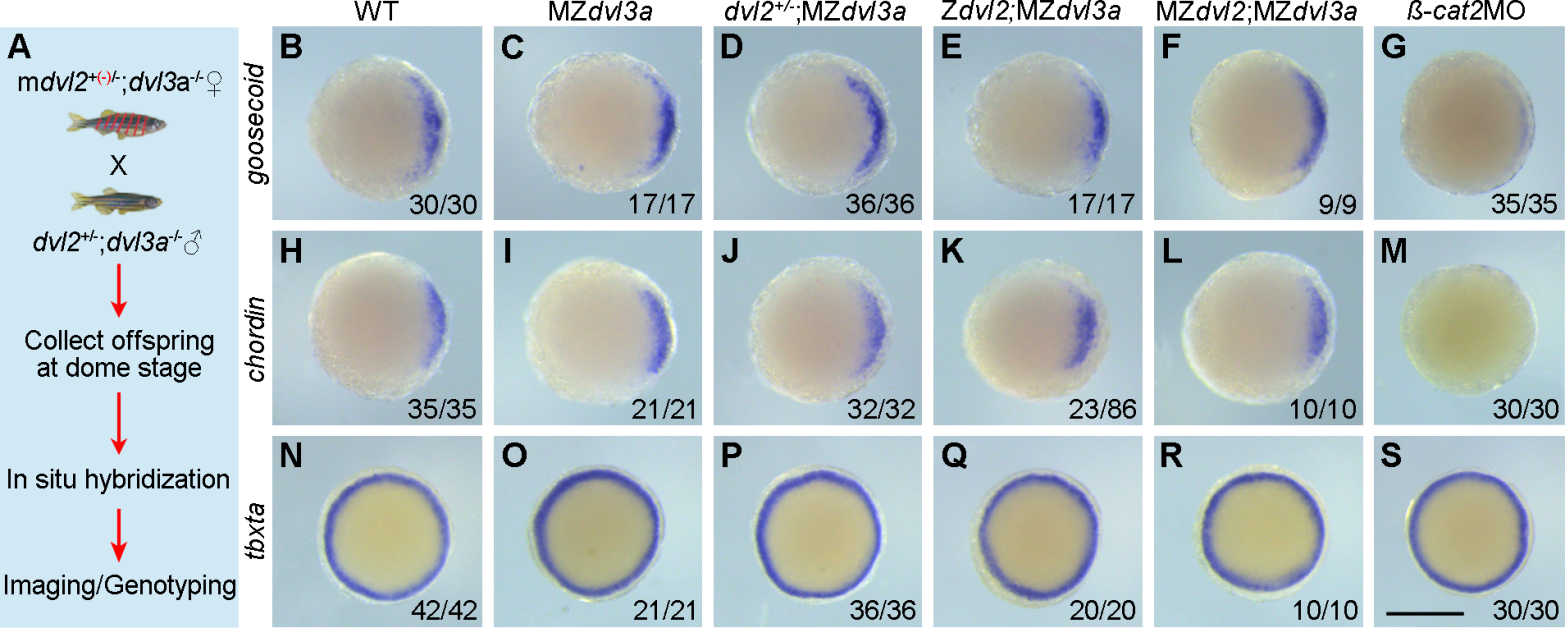
The expression of organizer genes is not affected in MZ*dvl2*;MZ*dvl3a* mutants. In situ hybridization analysis of the expression patterns of *goosecoid*, *chordin*, and *tbxta* at dome stage. Animal pole viewed embryos with dorsal region on the right. (A) Schematic representation for the analysis of MZ*dvl2*;MZ*dvl3a* embryos. (B-F) Representative images show similar expression patterns of *goosecoid* in WT embryos and in different mutants. (G) Knockdown of *ß-catenin2* strongly inhibits *goosecoid* expression. (H-L) Similar expression patterns of *chordin* in WT embryos and all indicated mutants. (M) Knockdown of *ß-catenin2* blocks *chordin* expression. (N-R) The expression patterns of *tbxta* are similar between WT embryos and different mutants. (S) Knockdown of *ß-catenin2* has no effect on *tbxta* expression. Scale bar: (B-J) 400 μm.

An implication of Dvl in activating maternal Wnt/ß-catenin signaling for dorsal fate specification has been unclear in *Xenopus* [17, 19, 20, 28], and this issue has not been addressed in zebrafish. Unlike the maternal effect mutant, *ichabod*, that reduces *ß-catenin2* transcripts and results in severe dorsal and anterior deficiencies during early development [37, 38], anterior structures such as eyes were present in MZ*dvl2*;MZ*dvl3a* mutants, although they were fused or cyclopic. This implies that dorsal and anterior fate specification should not be disrupted in the absence of maternal and zygotic Dvl2 and Dvl3a products. To further test this possibility, we examined the expression of dorsal mesoderm genes, *goosecoid* and *chordin*, and the pan-mesoderm gene, *tbxta* (*ntla*), at dome stage by in situ hybridization. Since only a small proportion of MZ*dvl2*;MZ*dvl3a* embryos was present in the offspring, and no phenotype difference could be observed among siblings at early stages, all the offspring from the crosses between female m*dvl2*^+(*-*)/-^;*dvl3a*^-/-^ and male *dvl2*^+/-^;*dvl3a*^-/-^ fish were collected, and divided into three parts for hybridization with each probe. Following in situ hybridization, the embryos were individually imaged and genotyped by sequencing (Fig 5A). The experiment was performed using three independent fish pairs and the results did not reveal any obvious difference in the expression patterns of *goosecoid*, *chordin*, and *tbxta* between WT embryos and MZ*dvl2*;MZ*dvl3a* mutants (Fig 5B–5F, 5H–5L and 5N–5R). By contrast, in the embryos injected with 2 ng *ß-catenin2* morpholino (*ß-cat2*MO), *goosecoid* expression was strongly reduced, *chordin* expression was absent, but *tbxta* expression was not affected (Fig 5G, 5M and 5S), confirming that maternal ß-catenin2 is required for dorsal fate specification. Together with phenotype analysis, this result suggests that maternal Dvl2 and Dvl3a may be not required for dorsal axis specification in zebrafish.

**Fig 6:**
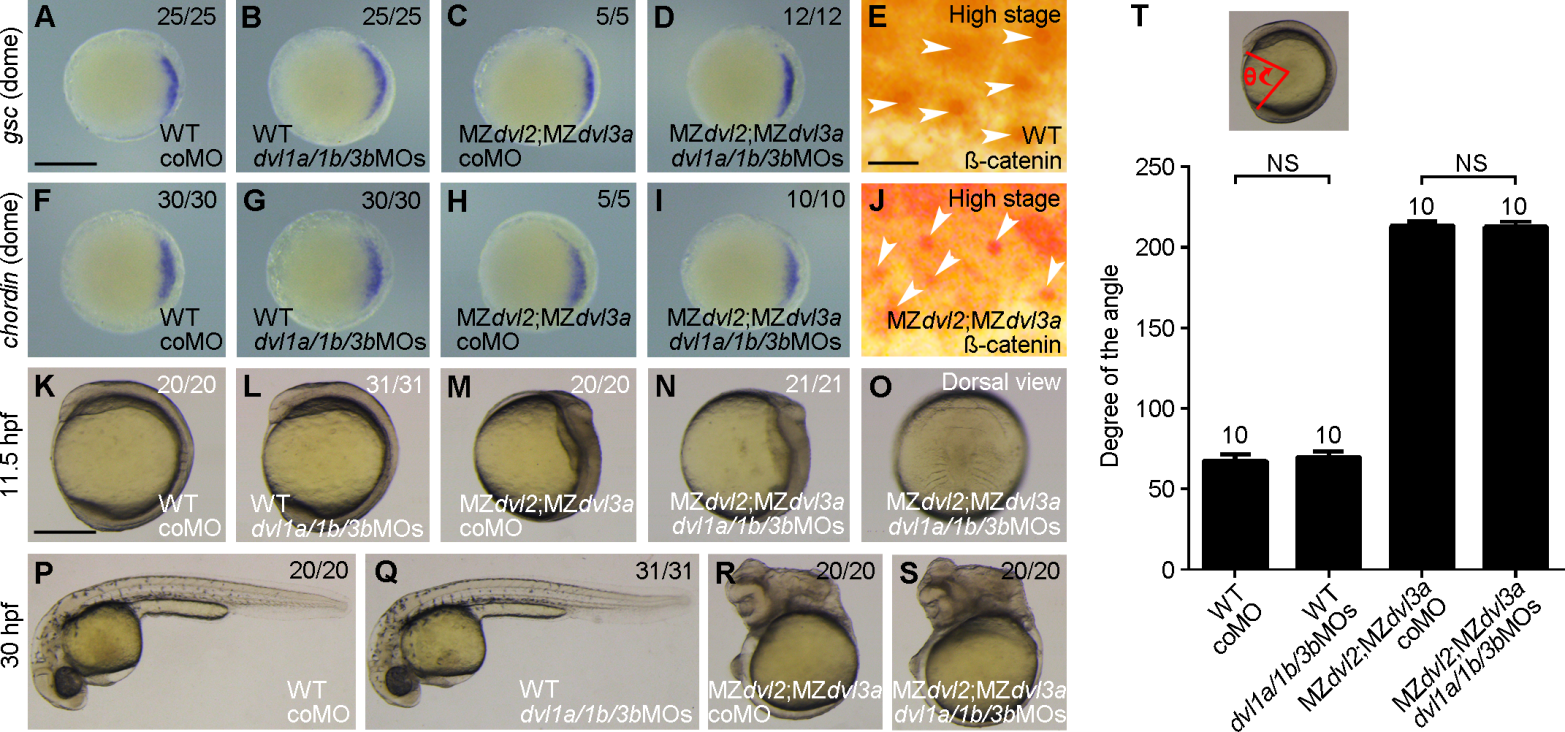
Knockdown of other *dvl* genes has no effect in MZ*dvl2*;MZ*dvl3a* mutants. The embryos were injected with coMO (2.5 ng) or a mixture of equal amount of *dvl1a*/*1b*/*3b*MOs (6 ng in total) at 1-cell stage, except for immunostaining. All mutant embryos were imaged after different analyses, followed by genotyping. (A-D) In situ hybridization analysis of *goosecoid* (*gsc*) expression in indicated embryos at dome stage. Animal pole view with dorsal region on the right. (E) Endogenous ß-catenin nuclear accumulation (arrows) in dorsal marginal cells of a WT embryo at high stage. (F-I) In situ hybridization analysis of *chordin* expression in indicated embryos at dome stage. Animal pole view with dorsal region on the right. (J) Endogenous ß-catenin nuclear accumulation (arrows) in dorsal marginal cells of an MZ*dvl2*;MZ*dvl3a* mutant at high stage. (K-O) Phenotypes of indicated embryos at 11.5 hpf. Lateral view, with a *dvl1a*/*1b*/*3b*MOs-injected MZ*dvl2*;MZ*dvl3a* embryo also shown in dorsal view (O). (P-S) Phenotypes of indicated embryos at 30 hpf. Lateral view, note that injection of *dvl1a*/*1b*/*3b*MOs does not change the phenotype of WT and MZ*dvl2*;MZ*dvl3a* embryos. (T) Statistical analysis of the extent of axis extension delay in indicated embryos at 11.5 hpf. Bars represent the mean ± s.d. from indicated numbers of embryos (NS, not significant). Scale bars: (A-D, F-I) 400 μm; (E, J) 25 μm; (K-S) 400 μm.

**Fig 7:**
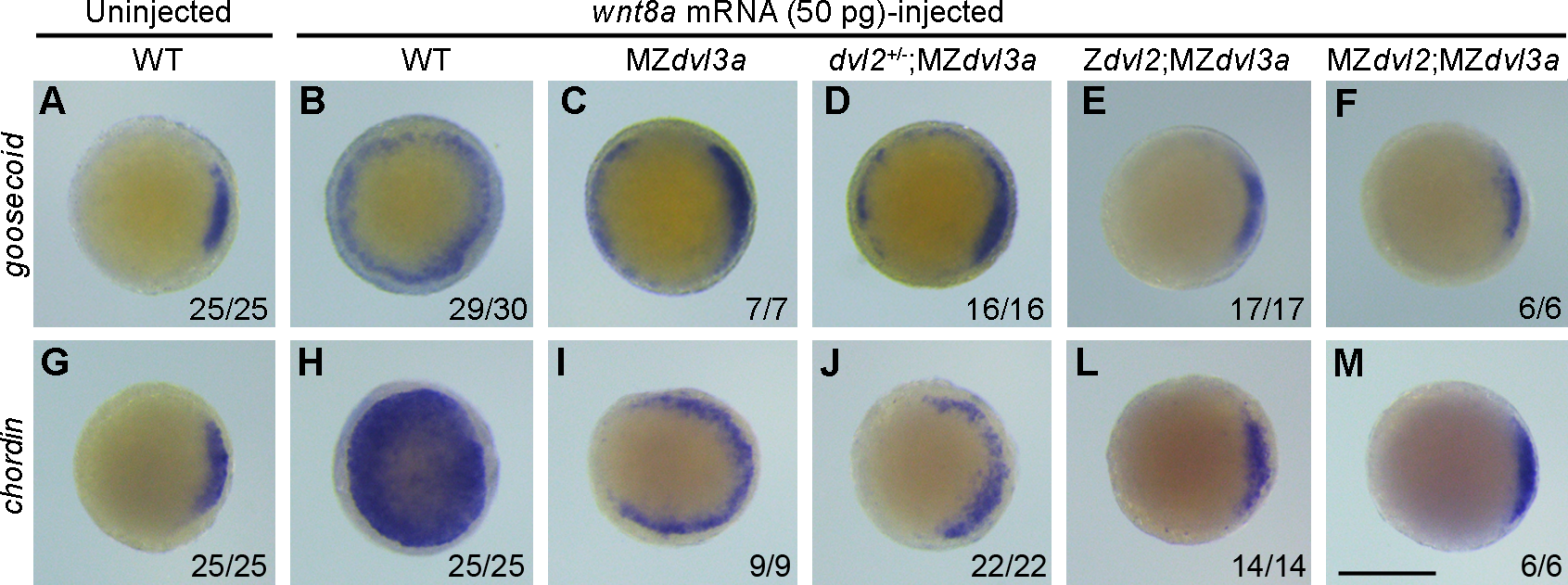
The inducing-activity of Wnt8a is blocked in MZ*dvl2*;MZ*dvl3a* mutants. In situ hybridization analysis of ectopic organizer gene expression at dome stage following Wnt8a overexpression in indicated embryos. Animal pole viewed embryos with dorsal region on the right. (A-F) *goosecoid* expression pattern in indicated embryos. (G-M) *chordin* expression pattern in indicated embryos. Notice that reduction of Dvl dosage progressively inhibits the inducing activity of Wnt8a, and MZ*dvl2*;MZ*dvl3a* embryos display a complete absence of ectopic *goosecoid* and *chordin* expression. All mutant embryos were derived from crosses between a female m*dvl2*^+(*-*)/-^;*dvl3a*^-/-^ and a male *dvl2*^+/-^;*dvl3a*^-/-^ fish, and were genotyped after in situ hybridization. Scale bar: 400 μm.

The negative result could be interpreted by the presence of residual amounts of other Dvl proteins. To test this possibility, we injected 2 ng of each *dvl1a*, *dvl1b*, and *dvl3b* morpholino oligonucleotides (referred to as *dvl1a*/*1b*/*3b*MOs) in 1-cell stage offspring derived from the crosses between female m*dvl2*^+(*-*)/-^;*dvl3a*^*-/-*^ fish and male *dvl2*^+/-^;*dvl3a*^*-/-*^ fish. In situ hybridization analysis of the expression of dorsal organizer genes *goosecoid* and *chordin* was performed at dome stage. The results showed that simultaneous knockdown of *dvl1a*, *dvl1b*, and *dvl3* in WT or MZ*dvl2*;MZ*dvl3a* embryos did not change the expression of *goosecoid* and *chordin*, compared to the embryos injected with 2.5 ng control morpholino (Fig 6A–6D and 6F–6I). Consistent with this observation, analysis by immunocytochemistry indicated that the nuclear accumulation of endogenous ß-catenin in dorsal marginal cells was comparable between WT and MZ*dvl2*;MZ*dvl3a* embryos at high stage (compare Fig 6E and 6J). Similarly, injection of *dvl1a*/*1b*/*3b*MOs had no effect on axis extension in WT embryos (Fig 6K, 6L, 6P, 6Q and 6T), and did not aggravate the defective phenotype of MZ*dvl2*;MZ*dvl3a* mutants (Fig 6M–6O and 6R–6T). Moreover, quantitative RT-PCR analysis at different stages, and analysis by RNA sequencing at 12 hpf all showed that there was no upregulation of *dvl1a*, *dvl1b* and *dvl3b* transcripts in MZ*dvl2*;MZ*dvl3a* mutants (S13 Fig). These results suggest that Dvl1a, Dvl1b, and Dvl3b could not compensate for the loss of Dvl2 and Dvl3a in early dorsal fate specification and in axis extension. To further confirm the absence of Dvl activity in MZ*dvl2*;MZ*dvl3a* early embryos, we injected synthetic *wnt8a* mRNA (50 pg) in 1-cell stage offspring obtained as above. In situ hybridization analysis of *goosecoid* and *chordin* ectopic expression clearly showed that overexpression of Wnt8a was able to strongly induce ectopic expression of these genes in WT embryos (Figs 7A, 7B, 7G and 6H). However, reducing Dvl dosage progressively decreased the activity of Wnt8a to activate ectopic organizer gene expression (Fig 7C, 7D, 7I and 7J). In particular, Wnt8a had no effect in Z*dvl2*;MZ*dvl3a* or MZ*dvl2*;MZ*dvl3a* embryos, which showed similar *goosecoid* and *chordin* expression patterns as in WT embryos (Fig 7E, 7F, 7L and 7M). The fact that Wnt8a failed to activate maternal Wnt/ß-catenin signaling leading to ectopic organizer gene expression suggests that Dvl activity should be absent in these mutants.

### Maternal Dvl2 and Dvl3a regulate AP patterning

Inhibition of zygotic Wnt/ß-catenin signaling, in particular Wnt8, is known to cause dorsalization and anteriorization [1, 3, 5]. However, this does not seem to occur in MZ*dvl2*;MZ*dvl3a* embryos, since the expression domains of *goosecoid* and *chordin* at 60% epiboly did not show obvious lateral expansion, although lateral and ventral expression of *axin2* was inhibited (S14 Fig). This suggests that downregulation of Wnt8 and Dvl activity exerts distinct effects to restrict the organizer domain.

**Fig 8:**
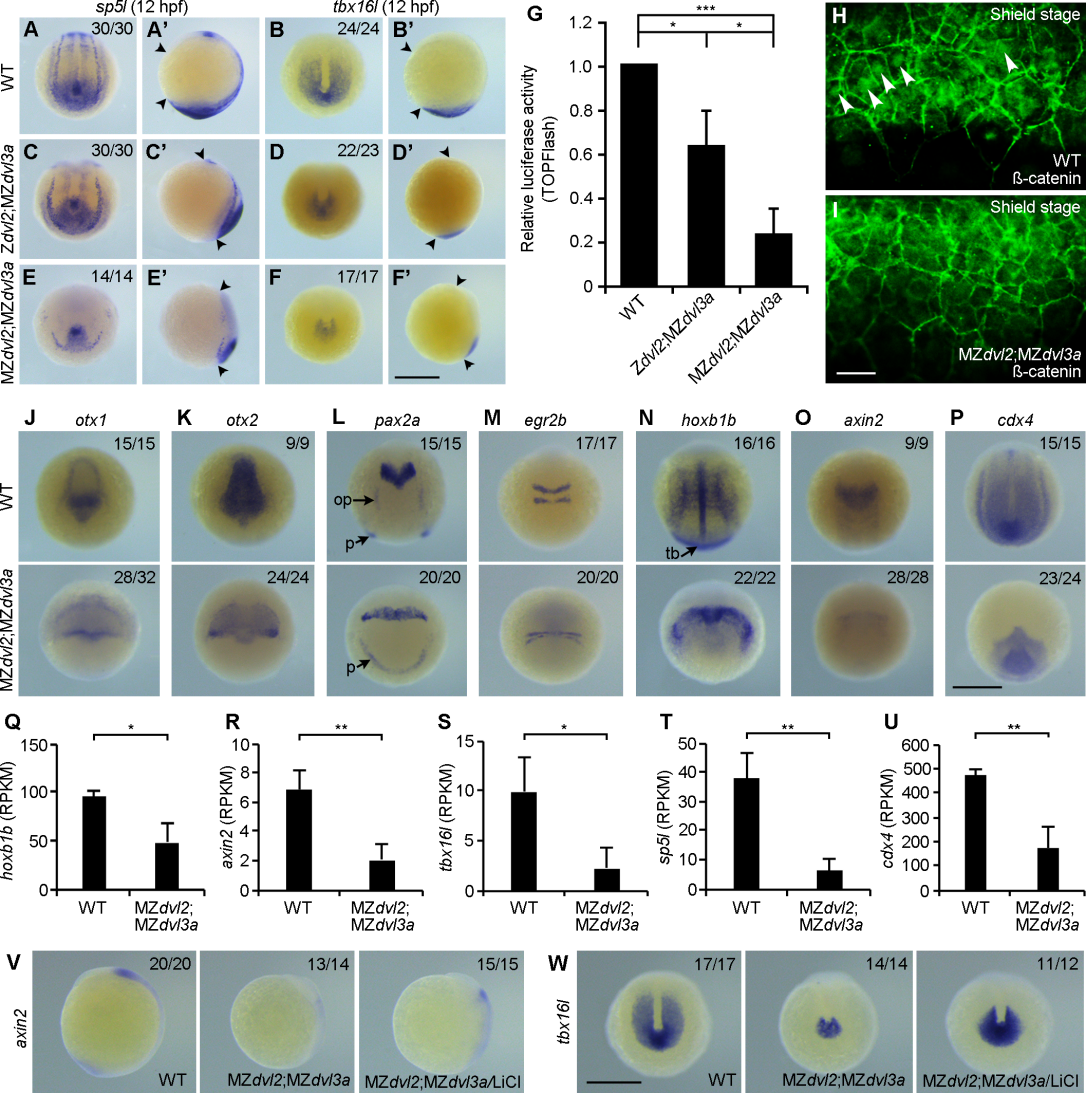
Cooperation of maternal and zygotic Dvl2 and Dvl3a in AP patterning. Mutant embryos were selected by the extent of CE defects at 12 hpf, and genotyped after in situ hybridization. Immunostaining was performed at shield stage, followed by genotyping. (A-B’) Expression patterns of *sp5l* and *tbx16l* in WT embryos. Arrowheads in lateral viewed embryos indicate the anterior end and posterior end. Other embryos are dorsal-posterior view. (C, C’) The expression of *sp5l* is not affected in Z*dvl2*;MZ*dvl3a* mutants. (D, D’) Strongly reduced *tbx16l* expression in Z*dvl2*;MZ*dvl3a* mutants. (E, E’) Strongly reduced *sp5l* expression in MZ*dvl2*;MZ*dvl3a* mutants. (F, F’) Residual expression of *tbx6l* in MZ*dvl2*;MZ*dvl3a* mutants. (G) TOPFlash luciferase reporter activity in Z*dvl2*;MZ*dvl3a* and MZ*dvl2*;MZ*dvl3a* mutants at 12 hpf. Bars represent the mean ± s.d. from three independent experiments (*, *P*<0.05; ***, *P*<0.001). (H) Endogenous ß-catenin nuclear accumulation (arrows) in ventral marginal cells of a WT embryo at shield stage. (I) Absence of ß-catenin nuclear accumulation in ventral marginal cells of an MZ*dvl2*;MZ*dvl3a* mutant at shield stage. (J-P) In situ hybridization analysis of indicated genes in WT and MZ*dvl2*;MZ*dvl3a* embryos at 12 hpf. Dorsal view with anterior to the top (J, K), dorsal view (L-O), and dorsal-posterior view (P). (R-U) Analysis of *hoxb1b* and zygotic Wnt/ß-catenin target genes by RNA-sequencing at 12 hpf. Note the significant decrease in RPKM (reads per kilobase million) for these genes in MZ*dvl2*;MZ*dvl3a* mutants. Bars represent the mean ± s.d. from three independent samples (*, *P*<0.05; **, *P*<0.01). (V, W) Lateral (V) and dorsal-posterior (W) views show rescue of *axin2* and *tbx16l* expression in MZ*dvl2*;MZ*dvl3a* mutants at 12 hpf, following LiCl (0.3 M) treatment for 8 minutes at 5 hpf. op, otic placode; p, pronephric mesoderm; tb, tailbud. Scale bar: (A-F’) 400 μm; (H, I) 20 μm; (J-P) 400 μm; (V, W) 400 μm.

To analyze how AP patterning is affected in MZ*dvl2*;MZ*dvl3a* mutants, we first performed in situ hybridization using two well characterized posterior markers, *sp5l* (*spr2*) and *tbx16l* (*tbx6l*), which mediate zygotic Wnt/ß-catenin signaling in posterior patterning [39–41]. At 12 hpf, the phenotypes specific to Z*dvl2*;MZ*dvl3a* or MZ*dvl2*;MZ*dvl3a* mutants were easily discernible, which allowed us to select sufficient mutant embryos from different crosses. Confirmed by genotyping after in situ hybridization, Z*dvl2*;MZ*dvl3a* mutants showed no obvious or only weak alternation in the expression of *sp5l* (Fig 8A, 8A’, 8C and 8C’), but they displayed a markedly reduced expression of *tbx16l* (Fig 8B, 8B’, 8D and 8D’). In MZ*dvl2*;MZ*dvl3a* mutants, however, the expression of *sp5l* was strongly decreased (Fig 8E and 8E’), and the expression of *tbx16l* was reduced to residual level (Fig 8F and 8F’). Consistently, TOPFlash luciferase assay revealed that there was approximately a 30% decrease of Wnt/ß-catenin transcriptional activity in Z*dvl2*;MZ*dvl3a* mutants at 12 hpf, and about a 75% decrease in MZ*dvl2*;MZ*dvl3a* mutants (Fig 8G). This decrease of reporter activity correlated well with a reduction of endogenous ß-catenin nuclear accumulation in ventral marginal cells at shield stage, when zygotic transcription has already started, as assayed by immunofluorescence staining (Fig 8H and 8I). These observations strongly suggest that maternal Dvl2 and Dv3a play an important role in the activation of zygotic Wnt/ß-catenin signaling, and that Dvl2 may exert a predominant role.

We further examined the expression of a panel of AP genes in MZ*dvl2*;MZ*dvl3a* mutants at 12 hpf by in situ hybridization. This indicated that the expression of *otx1* (forebrain and midbrain), *otx2* (forebrain, midbrain and midbrain-hindbrain boundary), and *pax2a* (midbrain-hindbrain boundary, otic placode and pronepheric mesoderm) was reduced, and widened mediolaterally due to impaired axis extension (Fig 8J–8L). The expression of *egr2b* (*krox20*) in rhombomeres 3 and 5, and *hoxb1b* in the notochord and paraxial mesoderm was compressed and widened (Fig 8M and 8N). Notably, the expression of *hoxb1b* in the tailbud was absent (Fig 8N). The expression of *axin2* in the neural plate, and of *cdx4* in the posterior paraxial mesoderm was strongly inhibited (Fig 8O and 8P). Analysis by RNA sequencing confirmed that *axin2*, *tbx16l*, *sp5l* and *cdx4*, which are zygotic Wnt/ß-catenin target genes in AP patterning [3, 39–41], as well as *hoxb1b*, were all downregulated (Fig 8Q–8U). These indicate that AP patterning, and in particular posterior development, are strongly affected in MZ*dvl2*;MZ*dvl3a* mutants.

To determine whether AP patterning defect in MZ*dvl2*;MZ*dvl3a* mutants was due to a decreased zygotic Wnt/ß-catenin signaling, offspring at 5 hpf derived from the crosses between female m*dvl2*^+(*-*)/-^;*dvl3a*^*-/-*^ fish and male *dvl2*^+/-^;*dvl3a*^*-/-*^ fish were treated with LiCl, which activates Wnt/ß-catenin signaling downstream of Dvl. In situ hybridization was performed to examine the expression of *axin2* and *tbx16l* at 12 hpf. Following genotyping, we found that MZ*dvl2*;MZ*dvl3a* mutants treated with LiCl displayed increased expression of *axin2* and *tbx16l*, compared to untreated mutants (Fig 8V and 8W). Injection of a low dose of synthetic mRNA (50 pg) encoding the constitutively active ß-catenin into 1-cell stage embryos also rescued the expression of *axin2* and *tbx16l*, but to a lesser extent (S15A–S15F Fig), likely due to the mosaic distribution of injected mRNA. However, phenotypic examination indicated that this injection could effectively rescue tail development in Z*dvl2*;MZ*dvl3a* mutants (S15G–S15I Fig). These observations further demonstrate that Dvl2 and Dvl3a deficiency causes defective AP patterning by preventing zygotic Wnt/ß-catenin signaling.

### Maternal Dvl2 and Dvl3a contribute to CE movements

**Fig 9:**
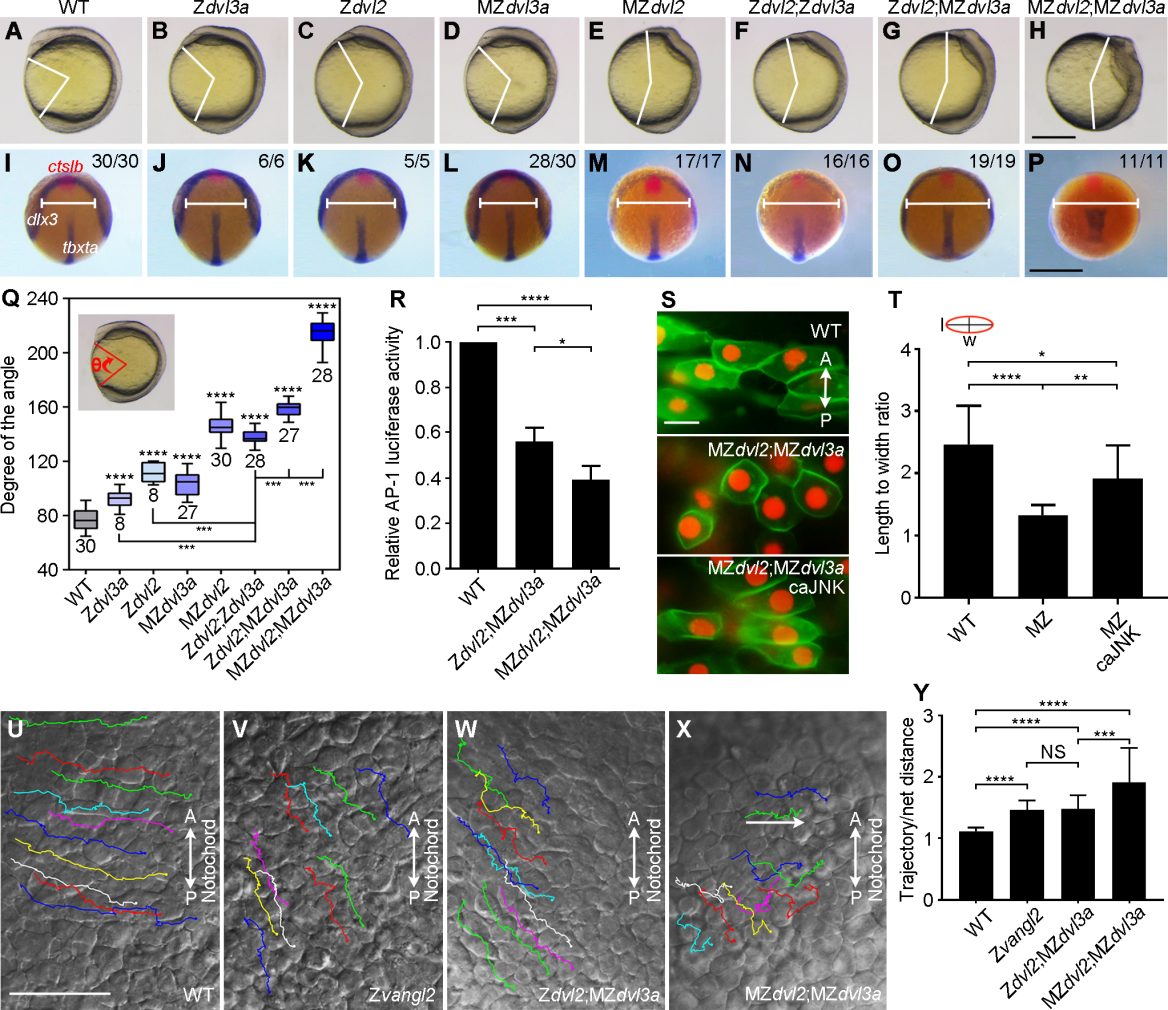
Dvl2 and Dvl3a dosages in CE movements and Wnt/PCP signaling activity. (A-H) Representative live images of WT and mutant embryos at 11.5 hpf. Lateral view, with anterior region on the top. (I-P) Dorsal view of indicated embryos simultaneously hybridized with *ctslb*, *dlx3*, and *tbxta* probes to reflect the position of prechordal plate mesoderm, neural plate borders, and notochord, respectively. MZ*dvl3a* mutants were from intercrosses between *dvl3a*^-/-^ carriers; MZ*dvl2* mutants were from crosses between female *dvl2*^-/-^ fish and male *dvl2*^+/-^ fish; Z*dvl2*;Z*dvl3a* mutants were from crosses between female *dvl2*^+/-^;*dvl3a*^-/-^ fish and male *dvl2*^+/-^;*dvl3a*^+/-^ fish; Z*dvl2*;MZ*dvl3a* mutants were from intercrosses between *dvl2*^+/-^;*dvl3a*^-/-^ carriers; MZ*dvl2*;MZ*dvl3a* mutants were from crosses between female m*dvl2*^+(-)/-^;*dvl3a*^-/-^ fish and male *dvl2*^+/-^;*dvl3a*^-/-^ fish. (Q) Statistical analysis shows that progressive reduction of Dvl2 and Dvl3a dosages increasingly aggravates axis extension defect. The embryos were imaged at 11.5 hpf, and genotyped before measuring the angle (inset). Bars represent the mean ± s.d. from indicated numbers of embryos collected from three independent experiments, and asterisks above the bars indicate significance with respect to WT embryos (***, *P*<0.001; ****, *P*<0.0001). (R) Reduced AP1 reporter activity in Z*dvl2*;MZ*dvl3a* and MZ*dvl2*;MZ*dvl3a* mutants at 12 hpf. Bars represent the mean ± s.d. from three independent experiments (*, *P*<0.05, ***, *P*<0.001, ****, *P*<0.0001). (S) Rescue of cell polarity of MZ*dvl2*;MZ*dvl3a* mutant cells by caJNK. Vertical bidirectional arrows indicate AP orientation. (T) Statistical analysis of the length (l) to width (w) ratio in indicated cells. Bars represent the mean ± s.d. from at least 10 cells in two representative images (*, *P*<0.05, **, *P*<0.01, ****, *P*<0.0001). (U-X) Still frames from live time-lapse images show the dorsal convergence and movement behaviors of lateral cells in indicated embryos (see also S1–S4 Movies). The trajectories of 10 randomly selected cells are traced. (Y) Statistical analysis of the ratio between the trajectory and the net mediolateral distance (as indicated by a horizontal arrow). Bars represent the mean ± s.d. from at least 15 cells in two or three representative images (***, *P*<0.001; ****, *P*<0.0001). Scale bars: (A-H) 400 μm; (I-P) 400 μm; (S) 20 μm; (U-X) 50 μm.

The most severely impaired axis elongation in MZ*dvl2*;MZ*dvl3a* mutants clearly suggests an important maternal contribution of Dvl2 and Dvl3a for CE movements. To further clarify this aspect and to determine Dvl dosages in Wnt/PCP signaling, we first compared the extent of axis extension defect between different mutants by phenotype analysis, and by simultaneous in situ hybridization using *tbxta* as a marker of the notochord, *dlx3* as a marker of the neural plate borders, and *ctslb* (*hgg1*) as a marker of the prechordal plate mesoderm. At 11.5 hpf, Z*dvl3a*, Z*dvl2* or MZ*dvl3a* mutants displayed weak, but obvious delay in neural plate convergence and notochord elongation (Fig 9A–9D, 9I–9L and 9Q). However, MZ*dvl2* mutants showed more severe defect (Fig 9E, 9M and 9Q). Thus, it is clear that either MZ*dvl2* or MZ*dvl3a* mutants present more severely affected CE phenotypes than the respective zygotic mutants. The same situation was also observed in double mutants. MZ*dvl2*;MZ*dvl3a* mutants displayed most severely impaired neural plate convergence and axis extension, compared with Z*dvl2*;Z*dvl3a* and Z*dvl2*;MZ*dvl3a* mutants (Fig 9F–9H and 9N–9Q), indicating that maternal Dvl dosage is important in Wnt/PCP signaling.

Dvl was shown to regulate cytoskeletal architecture and cell polarity upstream of Rac and Jun N-terminal kinase (JNK) both in *Xenopus* and zebrafish [42, 43]. When the AP1 luciferase reporter was used to monitor JNK activation [44], we found a 40% decrease of AP1 reporter activity in Z*dvl2*;MZ*dvl3a* mutants, and a 60% decrease in MZ*dvl2*;MZ*dvl3a* mutants (Fig 9R). Consistent with this result, analysis of cell polarity indicated that WT midline cells at 12 hpf were elongated mediolaterally, while MZ*dvl2*;MZ*dvl3a* mutant cells were rounded with a strongly reduced length to width ratio (Fig 9S and 9T), which was much similar as *dvl2* and *dvl3a* knockdown cells [43]. However, injection of synthetic mRNA (200 pg) encoding a constitutively active JNK, along with synthetic mRNAs encoding Histone2B and membrane GFP in one blastomere at 64-cell stage, significantly rescued mediolateral elongation of fluorescently-labeled descendent MZ*dvl2*;MZ*dvl3a* mutant cells (Fig 9S and 9T). This suggests that deficiency of Dvl2 and Dvl3a affects cell polarity by preventing at least partially JNK activation. Furthermore, we performed live time-lapse analysis of cell movement behaviors in Z*dvl2*;MZ*dvl3a* and MZ*dvl2*;MZ*dvl3a* mutants, in comparison with the PCP mutant *trilobite*/*vangl2* [45]. Since the AP axis was strongly shortened in these mutants, and the notochord became severely irregular, we examined the dorsal convergence behaviors of lateral cells. In WT embryos, these cells moved toward the notochord with regular trajectories (Fig 9U and S1 Movie). In zygotic *trilobite*/*vang2* mutants, lateral cells displaced along irregular trajectories and moved in more posterior direction (Fig 9V and S2 Movie). Lateral cells in Z*dvl2*;MZ*dvl3a* mutants moved similarly as in zygotic *trilobite*/*vang2* mutants (Fig 9W and S3 Movie). Strikingly, the directional movement of lateral cells in MZ*dvl2*;MZ*dvl3a* mutants was most severely affected. These cells moved along zigzagging trajectories, and displayed forward and back movements (Fig 9X and S4 Movie), with a more strong increase in the ratio of trajectory distance relative to net distance toward the notochord (Fig 9Y). These analyses strongly indicate an important maternal contribution of Dvl2 and Dv3a in Wnt/PCP signaling and CE movements. The fact that lateral cells in these mutants tend to move in more vegetal direction is likely due to an impaired AP patterning. Thus, it could not be excluded that the severely affected CE movements may result from the combined effects of defective Wnt/PCP and zygotic Wnt/ß-catenin signaling.

We further demonstrated the maternal contribution of Dvl2 and Dvl3a in axis extension using maternal double mutants. By crossing female m*dvl2*^+(*-*)/-^;*dvl3a*^-/-^ fish with male WT fish, the resulting double heterozygous embryos that carry a new mutation in one *dvl2* allele are maternal double mutants because of the absence of maternal Dvl2 and Dvl3a products (see S11 Fig). These M*dvl2*;M*dvl3a* mutants displayed strongly impaired axis extension at 11.5 hpf. However, they only presented a slightly shortened AP axis at 30 hpf (S16A–S16D and S16I Fig), suggesting that zygotic Dvl2 and Dvl3a can rescue axis extension at late stages. By contrast, M*dvl3a* mutants or *dvl2*^+/-^;M*dvl3a* mutants (lacking half of the maternal Dvl2 products), obtained from the crosses between female *dvl2*^+/-^;*dvl3a*^-/-^ fish and male WT fish, were either normal or displayed weak axis extension defect at 11.5 hpf, and were completely normal at 30 hpf (S16E–S16I Fig). These observations indicate a predominant contribution of maternal Dvl2 to embryonic axis elongation, and Dvl3a exerts a permissive effect. Altogether, our results show that both maternal and zygotic Dvl2 and Dvl3a cooperate to orchestrate CE movements and AP patterning.

## Discussion

Dvl proteins play key roles in both Wnt/ß-catenin and Wnt/PCP signaling pathways, and they are highly conserved and broadly expressed during early development in all vertebrates. However, many aspects of their involvement in early developmental processes remain elusive and enigmatic. In this study, we resolved some of the unanswered issues through comprehensive mutational analyses. Our results demonstrate that the two most abundantly expressed Dvl proteins, Dvl2 and Dv3a, are not required for early dorsal fate specification, which is dependent on the activation of maternal Wnt/ß-catenin signaling. Instead, maternal and zygotic Dvl2 and Dvl3a, in particular Dvl2, are important in CE movements, which are regulated by Wnt/PCP signaling, and in AP patterning. These findings help to clarify the implication of Dvl proteins in Wnt-regulated developmental events, and provide insight into the mechanisms underlying embryonic axis formation.

The early development in many vertebrates and invertebrates is supported by maternal products accumulated during oogenesis, zygotic transcription does not occur until the start of mid-blastula transition [1–5]. Inactivation of key genes implicated in early developmental processes frequently leads to embryonic lethality, or unproductive adults, preventing the analysis of maternal gene function. The situation becomes more complex when multiple gene paralogs are expressed. This is particularly true for functional analyses of Dvl paralogs during early development. Before this work, no *dvl* mutant has been reported in zebrafish, and the maternal and zygotic functions of Dvl proteins are not clear. We have used TALENs genome-editing technology to generate single mutants for all five zebrafish *dvl* paralogs, as well as a panel of *dvl2* and *dvl3a* triallelic and double homozygous mutants, and examined the maternal and zygotic contributions of Dvl2 and Dvl3a in embryonic patterning and morphogenetic movements. These mutants represent a valuable resource for the study of important developmental processes, which are dependent on the activation of the Wnt pathways.

A significant finding is the absence of implication for maternal Dvl2 and Dvl3a in early dorsal fate specification. This suggests that they are not required for the activation of maternal Wnt/ß-catenin signaling. Indeed, the expression of the dorsal organizer genes, *goosecoid* and *chordin*, is not affected in these mutants, whereas it is strongly decreased in *ß-catenin2* morphants. Moreover, the phenotypes of MZ*dvl2*;MZ*dvl3a* or M*dvl2*;M*dvl3a* mutants completely differ from those of zebrafish *ichabod* mutants, which display an absence of anterior structures caused by a reduced ß-catenin2 activity [37, 38]. This further argues against a requirement of Dvl2 and Dvl3a in dorsal axis formation. Our results from mutational analyses are consistent with the observations showing that simultaneous knockdown of *dvl2* and *dvl3a* in zebrafish does not apparently affect head formation [43, 46, 47], but mostly affects AP axis elongation and tail development [43]. They are supported by functional studies in *Xenopus*, which show that depletion of maternally expressed Dvl2 and Dvl3 from oocytes also has no effect on dorsal fate specification [19]. There is a possibility that these negative outcomes could be due to the inefficiency of the approaches to inhibit endogenous Dvl function [20]. However, our genetic evidence now suggests that the activation of maternal Wnt/ß-catenin signaling is independent of Dvl2 and Dvl3a. Although it was extremely difficult to assay maternal Wnt/ß-catenin activity in MZ*dvl2*;MZ*dvl3a* mutants at blastula stages, the correct expression of dorsal organizer genes is suggestive of an unaffected maternal Wnt/ß-catenin signaling.

Zebrafish genome contains at least five *dvl* genes, but transcriptomic analysis has revealed that *dvl2* and *dvl3a* represent approximately 98% of total *dvl* transcripts from fertilization until before the end of gastrulation [29], indicating that they are the major *dvl* genes expressed in the early embryo. It is unlikely that the loss-of-function of Dvl2 and Dvl3a could be compensated by other Dvl proteins, since they are expressed at an extremely low level, and their maternal and zygotic mutants do not result in any discernable phenotype at all stages examined. Moreover, our quantitative analyses indicate that the maternal and zygotic expression of *dvl1a*, *dvl1b* and *dvl3b* is not upregulated in MZ*dvl2*;MZ*dvl3a* mutants, and that simultaneous knockdown of these genes has no effect. A recent study indicates that Wnt/ß-catenin signaling is not affected in mouse ependymal cells lacking 5 of the 6 *Dvl* alleles [48], there is thus a possibility that trace amounts of Dvl protein could be sufficient for dorsal fate specification. However, several studies in zebrafish suggest a dose-dependent function of maternal Wnt/ß-catenin activity in organizer formation. In *ichabod* embryos with reduced ß-catenin2 level, dorsal and anterior deficiencies occur with variable expressivity [37], and knockdown of *ß-catenin2* increases the severity of *ichabod* phenotypes [38]. By contrast, MZ*dvl2*;MZ*dvl3a* embryos displayed correct organizer gene expression at the onset of zygotic transcription, suggesting that maternal Wnt/ß-catenin signaling should not be affected by the deficiency of Dvl activity. Thus, our present results support the model that early dorsal axis formation is a consequence of dorsal accumulation of ß-catenin caused by asymmetrical translocation of vegetally localized dorsal determinants [1–5]. They suggest that Dvls may be dispensable for the activation of maternal Wnt/ß-catenin signaling in dorsal fate specification. Nevertheless, there may be a possibility that maternal Wnt/ß-catenin signaling is activated by other mechanisms that are independent of maternal Wnt ligand/receptor signaling.

In *Xenopus*, Wnt11 was shown to be required for the activation of maternal Wnt/ß-catenin signaling in dorsal axis formation [49]. In zebrafish, vegetally localized maternal *wnt8a* is transported to the dorsal region and has been thought to play a role in specifying dorsal fate [50]. In this regard, it would be interesting to analyze the maternal effect following removal of the bicistronic *wnt8 locus* [51]. While this work was in progress, it was reported that maternal mutants for the two *wnt8a* open reading frames did not show axis formation defect [52]. Thus, our present results are consistent with this observation, and in particular, the trunk and posterior deficiencies in MZ*wnt8a* mutants are much similar as those observed in our MZ*dvl2*;MZ*dvl3a* mutants. However, it is worth to mention that MZ*wnt8a* mutants show dorsalized phenotype [52], whereas MZ*dvl2*;MZ*dvl3a* mutants exhibit CE defects with strongly reduced ventroposterior gene expression, without obvious dorsalizing effect. There are at least two credible explanations that may account for these differences. First, zygotic Wnt8a only activates Wnt/ß-catenin signaling and participates in ventral and posterior tissue formation, but not in CE movements. Second, extracellular Wnt8a ligand should also function to antagonize organizer-secreted Wnt inhibitors during gastrulation, and its absence leads to an expansion of organizer activity [5]. However, the absence of maternal and zygotic Dvl2 and Dvl3a should not disturb this functional antagonism, thus keeping early dorsal fate unaffected. Also as a consequence, MZ*dvl2*;MZ*dvl3a* mutants do not show anteriorization at late stages.

It is well established that Dvl plays a critical role in mediating the activation of Wnt/PCP signaling in CE movements during gastrulation [17]. Several studies in mice suggest that functional redundancy exists between Dvl proteins, and that the Wnt/PCP pathway during neurulation is more readily affected following reduction of Dvl dosage [25–27]. However, it is still unclear how Dvl dosage influences CE movements during gastrulation, and how Dvl proteins functionally interact in these processes in zebrafish. Our analyses by using single and double mutants clearly indicate that Dvl2 plays a predominant role in embryonic axis extension. There is only a partial functional redundancy between Dvl2 and Dvl3a, because MZ*dvl2* mutants display obvious CE defects, whereas MZ*dvl3a* mutants are phenotypically normal. Nevertheless, reducing Dvl3a dosage in zygotic *dvl2* mutants sensibly aggravates the defective CE phenotypes, indicating that Dvl3a exerts a permissive effect on Dvl2 in Wnt/PCP signaling. In mice, *Dvl2*^-/-^;*Dvl3*^+/-^ triallelic mutants exhibit more severely shortened AP axis than *Dvl3*^-/-^ or *Dvl2*^+/-^;*Dvl3*^-/-^ mutants [26]. Taken together, our present results suggest that Dvl2 plays an important role in Wnt/PCP signaling during CE movements, which may be conserved in vertebrates.

Interestingly, mutational analyses indicate that the absence of zygotic Dvl2 and Dvl3a function only results in moderate CE defects. However, removal of both maternal and zygotic Dvl2 and Dvl3a produces the most severely affected CE phenotypes, indicating clearly an important maternal contribution of these proteins in Wnt/PCP-mediated CE movements. This is further confirmed by analyzing the phenotypes of maternal *dvl2* and *dvl3a* double mutants, which show severe axis extension defect during gastrulation. Thus, by comparison of the extent of axis extension defect between Z*dvl2*;Z*dvl3a*, M*dvl2*;M*dvl3a*, and MZ*dvl2*;MZ*dvl3a* at different stages, it can be concluded that, to a large extent, maternal Dvl2 and Dvl3a may be sufficient to activate Wnt/PCP signaling in CE movements during gastrulation, whereas zygotic Dvl2 and Dvl3a are implicated to a lesser extent. Consistently, MZ*dvl2*;MZ*dvl3a* mutants display strongly reduced ability to activate the AP1 reporter, which monitors JNK activation [44], and the disrupted cell polarity can be rescued by a constitutively active JNK. However, the activity of the AP1 reporter was not completely blocked in MZ*dvl2*;MZ*dvl3a* mutants at late gastrula stages. This may be due to an independent activation of JNK signaling by other proteins such as the paraxial protocadherin that regulates morphogenesis and signals through the small GTPases RhoA and Rac1 to JNK [53, 54]. Altogether, our analyses indicate that Wnt/PCP-mediated CE movements are particularly sensitive to both maternal and zygotic Dvl dosages.

Another striking observation is the requirement for maternal Dvl function in AP patterning that is dependent on zygotic Wnt/ß-catenin signaling to activate region-specific gene expression. It is well established that an endogenous Wnt/ß-catenin signaling gradient, with highest activity in the posterior region, is important for AP patterning [3, 5]. At present, there is only limited evidence implicating Dvl in AP axis specification. In *Xenopus*, overexpression study shows that graded amounts of Dvl elicit AP fates in the prospective ectoderm [55], suggesting that Dvl dosage may be important to differentially activate zygotic gene expression along the AP axis. This is supported by the present observation showing an implication of Dvl2 and Dvl3a in this process in a dosage-dependent manner. We find that progressive reduction of Dvl dosage gradually elicits AP patterning defect, ranging from posterior deficiency to a complete lack of trunk and tail. The maternal contribution of Dvl2 and Dvl3a is clearly evident. While Z*dvl2*;Z*dvl3a* mutants display a relatively normal AP axis, Z*dvl2*;MZ*dvl3a* mutants begin to show caudal truncation. The most severe defect of AP patterning in MZ*dvl2*;MZ*dvl3a* mutants is clearly caused by a strongly impaired Wnt/ß-catenin signaling, which results in a severely decreased expression of target genes.

Maternal and zygotic Wnt/ß-catenin signaling has opposite functions in the specification of embryonic axes. While maternal Wnt/ß-catenin signaling specifies dorsal fate, zygotic Wnt/ß-catenin signaling induces ventroposterior mesoderm and inhibits anterior development [5]. This apparently contradicts with the requirement of maternal Dvl in AP axis specification, but it could be explained by the region-specific expression and regulation of other components of the Wnt/ß-catenin pathway. Following zygotic gene activation, the ventral region expresses several Wnt ligands. Thus, maternal Dvl proteins may serve to relay extracellular Wnt signals for region-specific activation of the pathway. Another possibility is that maternal Dvl may play a role in the specification of AP cell fates in the prospective ectoderm, as observed in *Xenopus* embryo [19, 55]. In overexpression experiments, high levels of Xdsh (Dvl2) activate posterior neural markers, whereas low levels induce the expression of anterior neural genes. Accordingly, in our zebrafish *dvl* mutants, deficiency of posterior and trunk tissues could be obtained by substantially reducing maternal Dvl dosage.

In the present study, we have revealed a predominant role for Dvl2 dosage both in CE movements and in AP patterning, however, it is clear that Dvl3a also cooperates with Dvl2 in these processes. Our results are consistent with previous studies indicating that Dvl proteins differentially activate the Wnt pathways and regulate distinct developmental processes. Indeed, the three mammalian DVL proteins differentially mediate the activation of Wnt/ß-catenin signaling in cultured cells [56]. In *Xenopus*, knockdown of *dvl1* and *dvl2* causes severe neural crest defects, while knockdown of *dvl3* affects muscle gene expression and sclerotome development [21]. In this regard, it is of interest to note that MZ*dvl2* mutants develop craniofacial defects that at least partially result from fusion or absence of neural crest-derived cartilages. In addition, the heart abnormality in *dvl2* and *dvl3a* double mutants is consistent with previous observations showing that *Dvl2* mutant mice display defects in cardiac neural crest development [25]. At present, it is still intriguing that why Dvl2 plays a major role both in Wnt/ß-catenin and Wnt/PCP signaling, and how it distinguishes these two pathways? Our previous structural and functional analyses have provided some clues as to how Dvl2 activity in Wnt/PCP signaling is regulated by its C-terminus [57, 58]. The present observations further indicate that the activaty of Wnt/ß-catenin or Wnt/PCP signaling during development may be regulated by Dvl dosages.

In summary, our findings have uncovered, to a significant extent, the manner in which these Dvl proteins are implicated in regulating the activation of different Wnt signaling pathways. In particular, we clarified that they are not required for dorsal fate specification, and demonstrated that maternal and zygotic Dvl2 dosages, in cooperation with Dvl3a, play a predominant role in regulating important zygotic events, such as AP patterning and morphogenetic movements.

## Materials and methods

### Ethical statement

All experiments using zebrafish adults and embryos were performed according to the ARRIVE guidelines and approved by the Ethics Committee for Animal Research of Life Science of Shandong University (permit number SYDWLL-2018-05).

### Zebrafish and microinjections

Zebrafish adult of the AB strain were maintained at 28.5°C. The embryos were staged as described [59], and for most experiments, were injected at 1-cell stage in the yolk using a PLI-100A Picoliter microinjector (Harvard Apparatus).

### Expression constructs and morpholino oligonucleotides

TALENs were assembled through Golden Gate Assembly [60], using the Golden Gate TALEN and TAL Effector Kit (cat#1000000016) from Addgene. TALEN repeat variable di-residues targeting sequences were cloned into modified pCS2+KKR and pCS2+ELD vectors [61]. Zebrafish *wnt8a* coding sequence was PCR-amplified and cloned in pCS2 vector. WT and mutant *dvl2* and *dvl3a* coding sequences were cloned in pCS2MT vector such that the proteins are C-terminally myc-tagged. Constructs for *Histone2B-RFP*, *mGFP*, *JNKK2-JNK1* (encoding a constitutively active Jun kinase) and Δ*N-ß-catenin* (encoding a constitutively active ß-catenin) have been previously described [43, 62–64]. Capped mRNAs were synthesized from linearized plasmids by in vitro transcription using appropriate RNA polymerases.

Translation-blocking morpholino antisense oligonucleotides against *ß-catenin2* [38], *dvl1a* (5′-AATCATTGACAGAAGAAGGAGCAAG-3′), *dvl1b* (5′-GGTATATGATTTTAGTCTCCGCCAT-3′), *dvl3b* (5′-TCTCCCTTCAGACAGCGACAATAAC-3′), and standard control morpholino (5′-CCTCTTACCTCAGTTACAATTTATA-3′) were synthesized by Gene Tools, and suspended in sterile water.

### Targeted gene mutations and generation of MZ*dvl2*;MZ*dvl3a* mutants

The two TALEN mRNAs were mixed at equal amounts (200 pg each) and injected into 1-cell stage embryos. The targeting efficiency was determined by Sanger sequencing of PCR products amplified from genomic DNA extracted from 15 randomly selected F0 embryos at 24 hpf. When the result indicates a positive targeting effect, other embryos were reared to adulthood for outcross to screen F1 heterozygotes using genomic DNA extracted from the tail fin.

To generate MZ*dvl2*;MZ*dvl3a* mutant lines, we first used germline replacement approach by transplanting blastoderm cells from Z*dvl2* donors at dome stage into *dvl2*^+/-^;*dvl3a*^-/-^ hosts. Since this only generated 21 male chimera fish, we next used a strategy to target the remaining *dvl2* WT allele in *dvl2*^+/-^;*dvl3a*^-/-^ embryos. The offspring obtained from crosses between *dvl2*^+/-^;*dvl3a*^-/-^ carriers were injected at 1-cell stage with *dvl2* TALEN mRNAs (100 pg each) and raised to adulthood. Due to the mosaic distribution of TALEN mRNAs and incomplete targeting efficiency, mosaic fish with mixed *dvl2*^+/-^;*dvl3a*^-/-^ and *dvl2*^-/-^;*dvl3a*^-/-^ genotypes could be obtained, and denoted as m*dvl2*^+(*-*)/-^;*dvl3a*^-/-^, for mosaic homozygous *dvl2* mutations. Female m*dvl2*^+(*-*)/-^;*dvl3a*^-/-^ fish were then crossed with male *dvl2*^+/-^;*dvl3a*^-/-^, and the resulting offspring were screened by PCR using allele-specific primers (S1 Table), followed by sequencing to detect *de novo* mutations in the *dvl2* allele. The offspring that contained a new indel along with the original indel were MZ*dvl2*;MZ*dvl3a* mutants, and the parental fish were selected for further experiments. To obtain M*dvl2*;M*dvl3a* mutants, female m*dvl2*^+(-)/-^;*dvl3a*^-/-^ fish were crossed with male WT fish. The resulting *dvl2* and *dvl3a* heterozygous offspring that carry either the original indel or a new indel in one *dvl2* allele were devoid of maternal Dvl2 and Dvl3a gene products.

### Whole-mount in situ hybridization and immunostaining

Whole-mount in situ hybridization was performed as previously described [65]. The constructs for *goosecoid*, *chordin*, *tbxta*, *dlx3*, *ctslb*, *otx2*, *pax2a* and *egr2b* were reported previously [65, 66], and *otx1*, *axin2*, *hoxb1b*, *cdx4*, *sp5l*, *tbx16l* and *dvl2* constructs were generated by cloning PCR framents in pZeroBack/Blunt Vector (Tiangen). They were labeled using digoxigenin-11-UTP (Roche Diagnostics). Staining of embryos simultaneously hybridized with *tbxta*, *dlx3*, and *ctslb* probes was performed using NBT/BCIP and Fast Red as substrates (Roche Diagnostics), respectively.

For immunostaining, the embryos were fixed in 4% paraformaldehyde at 4°C overnight, and washed with PBST (PBS, 0.1% Triton X-10), they were then incubated in mouse monoclonal anti-ß-catenin antibody (1/250, Sigma-Aldrich, C7207) at 4°C overnight. After several washes in PBST, embryos at high stage were incubated with horseradish peroxidase conjugated secondary antibody (1/500, INTERCHIM), followed by incubation in diaminobenzidine substrate, and embryos at shield stage were incubated with Alexa-488 conjugated secondary antibody (1/1000, INTERCHIM), followed by confocal microscopic imaging (Zeiss, LSM700).

### Cartilage staining

Larvae at 5 dpf were fixed in 4% paraformaldehyde at 4°C overnight, then washed twice in PBS for 10 minutes. The larvae were incubated in alcian blue solution (0.37% HCl, 70% ethanol, 0.1% alcian blue) for 6 hours, and washed in destaining solution (1% HCl, 70% ethanol). Following dehydration in ethanol, the larvae were cleared in benzyl benzoate, and imaged using an upright microscope (Leica DM2500).

### Live time-lapse imaging

Embryos at 90% epiboly stage were mounted in a cavity microscope slide in 1% low-melting agarose as described [43]. Cell movements were recorded using an upright light microscope (Leica, LM2500) equipped with a CCD digital camera (Leica, IC180), under differential interference contrast. The embryos were imaged every 30 seconds for a period of 60 minutes, and mutant embryos were then subjected to genotyping by Sanger sequencing. Time-lapse movies were generated using ImageJ software (NIH Image).

### Analysis of cell polarity

At 64-cell stage, a single marginal cell was injected with a mixture of *Histone2B-RFP* (50 pg) and *mGFP* (100 pg) mRNAs, with or without *caJNK* mRNA (200 pg). At 12 hpf, mosaically labeled embryos were dechorionated and placed on a microscope slide in a drop of Ringer’s solution. The yolk was removed, and the embryos were flat mounted with neuroectoderm facing upward. Following image acquisition using an upright fluorescence microscope (Leica LM2500), the embryos were subjected to genotyping.

### Semi-quantitative and quantitative RT-PCR, and RNA sequencing

Total RNA was reverse transcribed using M-MLV reverse transcriptase (Invitrogen). Semi-quantitative PCR was performed using gene-specific primers, with ß-actin as a loading control (S1 Table). The intensity of PCR products was analyzed using the Lane 1D software (Sagecreation). Quantitative PCR was performed using Quant qRT-PCR Kit (Tiangen) with gene-specific primers (S1 Table). RNA sequencing was performed on Illumina HiSeq 2000, using 12 hpf mRNA libraries constructed by TruSeq RNA Library Preparation Kit. The data were aligned and analyzed as described [65].

### Western blotting

Zebrafish embryos were lysed in ice-cold lysis buffer (100 mM NaCl, 10 mM Tris-HCl, pH 7.5, 5 mM EDTA, 1% Triton X-100) containing 1 x protease inhibitor cocktail (Sigma-Aldrich). The samples were separated by polyacrylamide gel electrophoresis, transferred to nitrocellulose membrane, probed with anti-myc (1/1000, Santa Cruz Biotechnology) and anti-α-tubulin (1/1000, GeneTex, GTX124303) antibodies, and detected using the Western-Lightning Plus-ECL substrate (PerkinElmer).

### PCR-based genotyping

Single embryo was placed in an Ependorf tube containing 40 μl of lysis buffer (10 mM Tris-HCl, pH 8.0, 2 mM EDTA, 0.2% Triton X-100, 100 μg/ml proteinase K), and homogenized by pipetting. The tube was heated at 50°C for 2 hours, then at 94°C for 10 minutes. After a brief centrifugation, 1 μl of the solution was used for PCR reaction.

### Luciferase assays

WT and mutant embryos at 1-cell stage were injected with 50 pg TOPFlash or AP1 reporter DNA, along with 5 pg pRL-TK DNA as an internal control. Fifteen to twenty embryos at 12 hpf were manually dechorionated and lysed in 60 μl lysis buffer (Promega). The lysate was clarified by centrifugation and luciferase activities were measured using the Dual-Luciferase® Reporter Assay System (Promega), according to the manufacturer’s instruction. The values were normalized with respect to Renilla luciferase activities, and the value in control condition was set as 1, and expressed as relative luciferase activity.

### Statistical analysis

All data were obtained from at least three independent experiments, and analyzed using paired Student’s *t* test.

## Supporting information

S1 Fig Targeted mutation of *dvl1a* gene. (A) TALENs-targeted sites (red) in the seventh exon and adjacent intron. Nucleotides in italic indicate intron sequence. (B) A deletion of 201 nucleotides in the seventh exon and the adjacent intron. Dots are introduced in WT sequence to optimize alignment, and dashes represent deleted nucleotides. (C) Schematic of Dvl domains shows truncated Dvl1a protein.
(JPG)

S2 Fig Targeted mutation of *dvl1b* gene. (A) TALENs-targeted sites (red) in the first exon. (B) A deletion of 8 nucleotides (dashes) within the exon. (C) Schematic of Dvl domains shows truncated Dvl1b protein.
(JPG)

S3 Fig Targeted mutation of *dvl2* gene. (A) TALENs-targeted sites (red) in the first exon and adjacent intron. Nucleotides in italic indicate intron sequence. (B) A deletion of 5 nucleotides (dashes) in the first exon. (C) Schematic of Dvl domains shows truncation of Dvl2 protein.
(JPG)

S4 Fig Targeted mutation of *dvl3a* gene. (A) TALENs-targeted sites (red) in the first exon. (B) A deletion of 5 nucleotides (dashes) within the exon. (C) Schematic of Dvl domains shows truncation of Dvl3a protein.
(JPG)

S5 Fig Targeted mutation of *dvl3b* gene. (A) TALENs-targeted sites (red) in the first exon. (B) An insertion of 13 nucleotides (lowercases) within the exon. Dots are introduced in WT sequence to optimize alignment. (C) Schematic of Dvl domains shows truncated Dvl3b protein.
(JPG)

S6 Fig Phenotypes of MZ*dvl1a*, MZ*dvl1b* and MZ*dvl3b* mutants at different stages. (A-C) WT embryos. (D-F) MZ*dvl1a* mutants. (G-I) MZ*dvl1b* mutants. (J-L) MZ*dvl3b* mutants. All embryos are lateral view. The anterior region of 11.5 hpf embryos is positioned on the top. Scale bars: (A, D, G, J) 400 μm; (B, E, H, K) 400 μm; (C, F, I, L) 400 μm.
(JPG)

S7 Fig Relative abundance of *dvl* transcripts during cleavage and gastrula stages. The graph was obtained by analyzing published RNA-seq data (Harvey et al., 2013. See reference 29 in the main text). FPKM, fragments per kilobase million.
(JPG)

S8 Fig Genotyping of *dvl2* and *dvl3a* mutants by allele-specific PCR. Shown are the PCR products that should be amplified from genomic DNA in WT, heterozygous, and homozygous adult fish, by using allele-specific primers (see S1 Table for primer sequences).
(JPG)

S9 Fig Analysis of mutant *dvl2* and *dvl3a* transcripts. (A) Semi-quantitative RT-PCR analysis to detect mutant *dvl2* and *dvl3a* transcripts at 1-cell stage. *ß-actin* served as a loading control. NMD can be observed for *dvl2* and *dvl3a* transcripts, respectively. (B, C) Quantification of mutant *dvl2* and *dvl3a* mRNA levels in M*dvl2* and MZ*dvl3a* mutants. The expression level in WT embryo is set as 1 after normalization with *ß-actin*. Bars represent the mean ± s.d. from three experiments (*, *P*<0.05; ****, *P*<0.0001). (D, E) In situ hybridization analysis of *dvl2* transcripts in WT and M*dvl2* embryos. RT, reverse transcriptase.
(JPG)

S10 Fig Western blotting analysis of mutant Dvl2 and Dvl3a proteins. Synthetic mRNAs encoding C-terminally myc-tagged WT and mutant Dvl2 and Dvl3a were expressed in zebrafish embryos. (A, B) Western blotting shows that mutant *dvl2* and *dvl3a* transcripts are not translated.
(JPG)

S11 Fig Schematic representation of the occurrence of MZ*dvl2*;MZ*dvl3a* and M*dvl2*;M*dvl3a*. (A) In the crosses between a mosaic female m*dvl2*^+(*-*)/-^;*dvl3a*^-/-^ fish and a male *dvl2*^+/-^;*dvl3a*^-/-^ fish, when the germline of the female fish contains a novel mutation for *dvl2*, the gametes that it produces will give rise to an MZ*dvl2*;MZ*dvl3a* offspring when fertilized by a male gamete with the original mutation, and an M*dvl2*;MZ*dvl3a* offspring when fertilized by a male gamete with WT *dvl2* allele. The same female gametes will give rise to an M*dvl2*;M*dvl3a* offspring when fertilized by a male gamete from WT fish. (B) When a female m*dvl2*^+(*-*)/-^;*dvl3a*^-/-^ fish is crossed with a male *dvl2*^+/-^;*dvl3a*^-/-^ fish, if the germline of the female fish only contains the original mutation, the genotypes of offspring should include *dvl2*^+/-^;MZ*dvl3a*^-/-^, MZ*dvl3a*, or Z*dvl2*;M*dvl3a* zygotes, depending on the genotype of the male gamete. These female gametes will give rise to M*dvl3a* offspring when fertilized by a male gamete from WT fish. In all these cases, the early embryos should lack half of the *dvl2* gene product. Only the *dvl2* alleles are indicated in the schema.
(JPG)

S12 Fig Development of cyclopia in *dvl2* and *dvl3a* mutants. (A-E) Ventral view of representative images of normal and different degrees of eye phenotypes at 3 dpf. (F) Quantitative analysis of different degrees of eye phenotypes in indicated mutants. Except for WT embryos, all mutants were analyzed from three independent crosses using the same fish pair (indicated below the horizontal line). Numbers on the top of each column indicate total embryos carrying the indicated genotypes (above the horizontal line). Scale bar: (A-E) 400 μm.
(JPG)

S13 Fig Analysis of *dvl1a*, *dvl1b*, and *dvl3b* transcripts in MZ*dvl2*;M*dvl3a* mutants. (A-I) Quantitative RT-PCR analysis of *dvl1a* (A, D, G), *dvl1b* (B, E, H), and *dvl3b* (C, F, I) at 1-cell stage (A-C), 50% epiboly (D-F) and 12 hpf (G-I). The expression level in WT embryo is set as 1 after normalization with *ß-actin*, and bars represent the mean ± s.d. from three independent experiments (NS, not significant). (J-L) Analysis of *dvl1a* (J), *dvl1b* (K), and *dvl3b* (L) expression levels by RNA sequencing at 12 hpf. Bars represent the mean ± s.d. from three independent samples (NS, not significant). RPKM, reads per kilobase million
(JPG)

S14 Fig Absence of dorsalization in MZ*dvl2*;MZ*dvl3a* mutants. In situ hybridization analysis of dorsoventral markers. (A, B) The expression of *axin2* at 50% epiboly is inhibited in MZ*dvl2*;MZ*dvl3a* mutants. (C-F’) The expression domains of *goosecoid* (*gsc*) and *chordin* in MZ*dvl2*;MZ*dvl3a* mutants at 60% epiboly do not show ventral expansion, but reflect impaired AP extension. Scale bar: 400 μm.
(JPG)

S15 Fig Overexpression of ß-catenin partially rescues AP patterning in MZ*dvl2*;MZ*dvl3a* mutants. Embryos at 1-cell stage derived from crosses between female m*dvl2*^+(*-*)/-^;*dvl3a*^-/-^ fish and male *dvl2*^+/-^;*dvl3a*^-/-^ fish were injected with Δ*N-ß-catenin* mRNA (50 pg). Following in situ hybridization or phenotype analysis, the embryos were subjected to genotyping. (A-F) ΔN-ß-catenin partially rescues *axin2* and *tbx16l* expression in MZ*dvl2*;MZ*dvl3a* mutants. Arrowheads indicate the *axin2* anterior expression domain. (G-I) ΔN-ß-catenin partially rescues tail development in Z*dvl2*;MZ*dvl3a* mutants (arrow). Scale bar: (A-F) 400 μm; (G-I) 400 μm.
(JPG)

S16 Fig Maternal contribution of Dvl2 and Dvl3a in axis extension. The embryos were imaged at 11.5 hpf and 30 hpf, followed by genotyping, and the extent of axis extension defect was reflected by the angle between the anterior end and the posterior end at 11.5 hpf. (A, B) WT embryos. (C, D) M*dvl2*;M*dvl3a* mutants from crosses between female m*dvl2*^+(*-*)/-^;*dvl3a*^-/-^ fish and male WT fish were genotyped for the presence of a novel indel (red) along with a WT allele in the *dvl2* locus. They have *dvl2* and *dvl3a* heterozygous mutations. (E, H) The offspring with the two possible genotypes (*dvl2*^+/+^;*dvl3a*^+/-^ and *dvl2*^+/-^;*dvl3a*^+/-^; the effects of these mutations are indicated in parenthesis), derived from a cross between female *dvl2*^+/-^;*dvl3a*^-/-^ fish and male WT fish, are maternal mutants for *dvl3a*, with a reduced dosage of maternal *dvl2* in both cases, despite of the genotype. (I) Statistical analysis of the extent of axis extension delay in three types of maternal mutants. Bars represent the mean ± s.d. from indicated numbers of embryos, and asterisks above the bars show significance with respect to WT embryos (***, *P*<0.001; ****, *P*<0.0001). (J) Quantitative analysis of defective axis extension at 11.5 hpf. Each type of cross was done using three independent fish pairs, and total numbers of embryos analyzed are indicated on the top of each column. Subjective measures of axis extension defect are shown on the embryos at 11.5 hpf. Scale bar: (A, C, E, G) 400 μm; (B, D, F, H) 400 μm.
(JPG)

S1 File Numerical data. This file contains statistical data corresponding to all graphs presented in the manuscript.
(ZIP)

S1 Table Primers for semi-quantitative PCR, quantitative PCR and allele-specific PCR. (DOCX)

S1 Movie Dorsal convergence of lateral cells in a WT embryo. Time-lapse recording of cell movement toward the notochord (right side) was performed at 90% epiboly.
(AVI)

S2 Movie Dorsal convergence of lateral cells in a zygotic *trilobite*/*vangl2* embryo. Time-lapse recording of cell movement toward the notochord (right side) was performed at 90% epiboly.
(AVI)

S3 Movie Dorsal convergence of lateral cells in a Z*dvl2*;MZ*dvl3a* embryo. Time-lapse recording of cell movement toward the notochord (right side) was performed at 90% epiboly.
(AVI)

S4 Movie Dorsal convergence of lateral cells in an MZ*dvl2*;MZ*dvl3a* embryo. Time-lapse recording of cell movement toward the notochord (right side) was performed at 90% epiboly.
(AVI)
