# Supplementary figures and images for: Mutational analysis of dishevelled genes in zebrafish reveals distinct functions in embryonic patterning and gastrulation cell movements
Source: PLoS Genet. 2018 Aug 6;14(8):e1007551. doi: 10.1371/journal.pgen.1007551 (PMC6095615; doi:10.1371/journal.pgen.1007551)

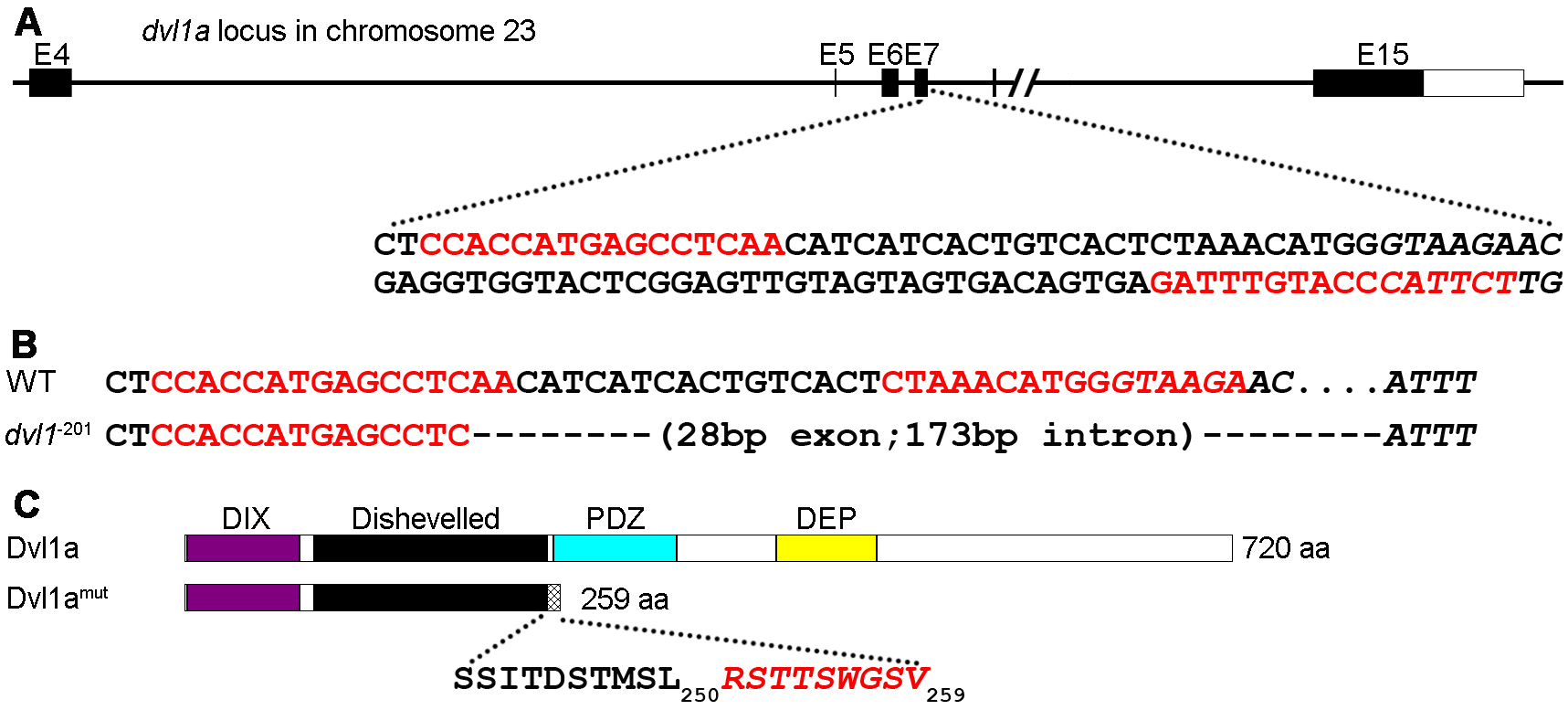

Supplement: S1 Fig — (A) TALENs-targeted sites (red) in the seventh exon and adjacent intron. Nucleotides in italic indicate intron sequence. (B) A deletion of 201 nucleotides in the seventh exon and the adjacent intron. Dots are introduced in WT sequence to optimize alignment, and dashes represent deleted nucleotides. (C) Schematic of Dvl domains shows truncated Dvl1a protein. (JPG) [file pgen.1007551.s001.jpg]

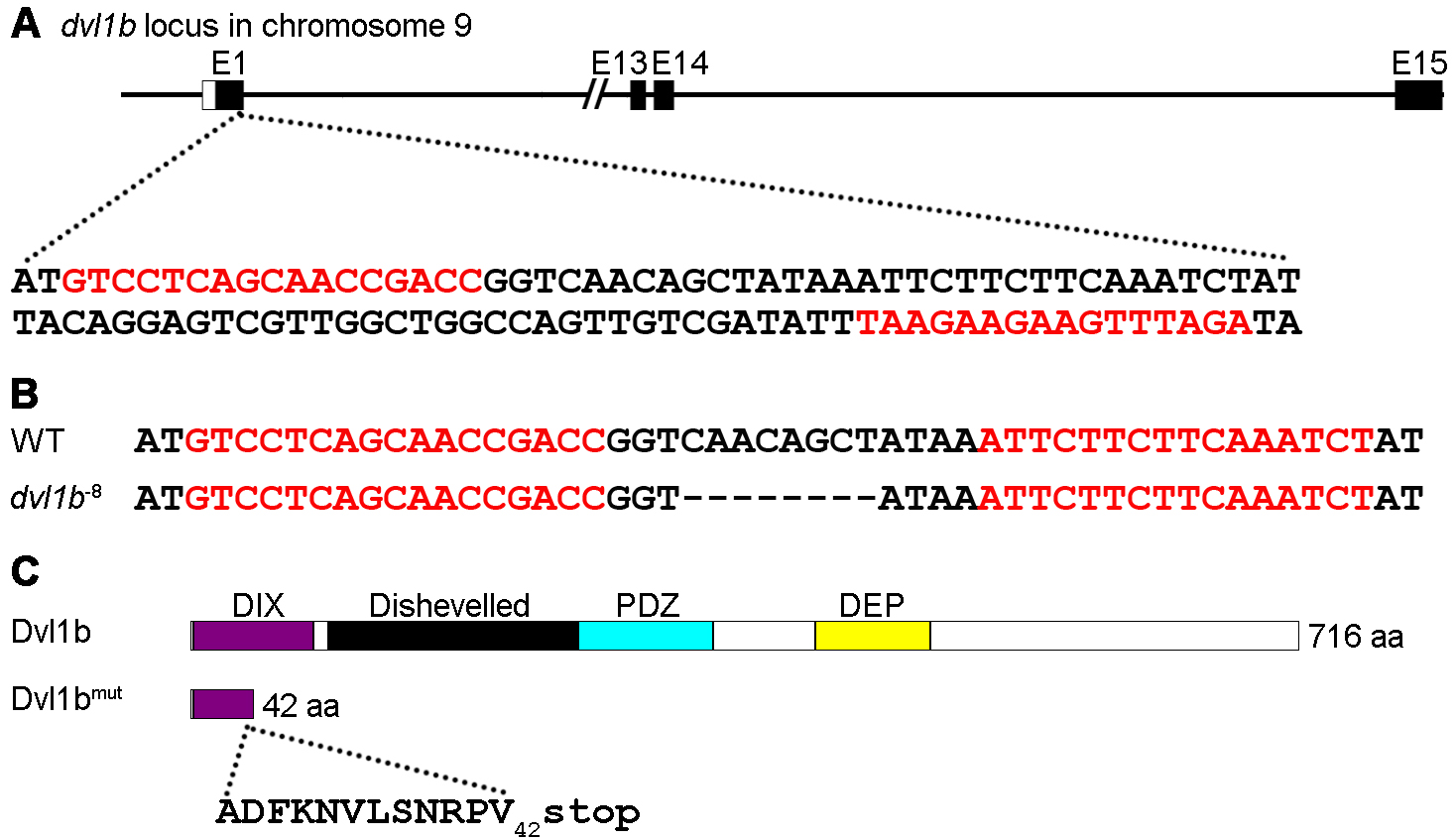

Supplement: S2 Fig — (A) TALENs-targeted sites (red) in the first exon. (B) A deletion of 8 nucleotides (dashes) within the exon. (C) Schematic of Dvl domains shows truncated Dvl1b protein. (JPG) [file pgen.1007551.s002.jpg]

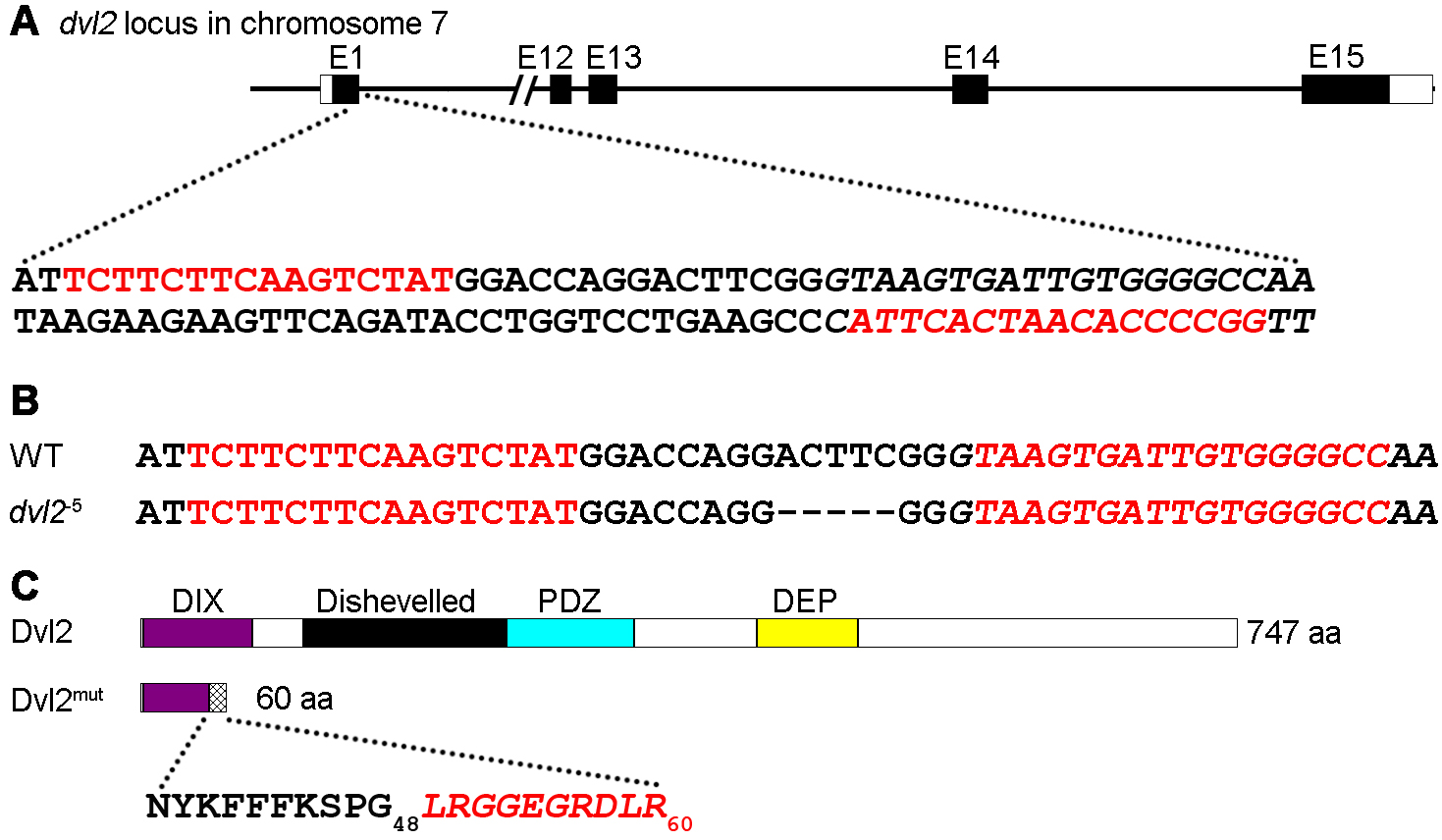

Supplement: S3 Fig — (A) TALENs-targeted sites (red) in the first exon and adjacent intron. Nucleotides in italic indicate intron sequence. (B) A deletion of 5 nucleotides (dashes) in the first exon. (C) Schematic of Dvl domains shows truncation of Dvl2 protein. (JPG) [file pgen.1007551.s003.jpg]

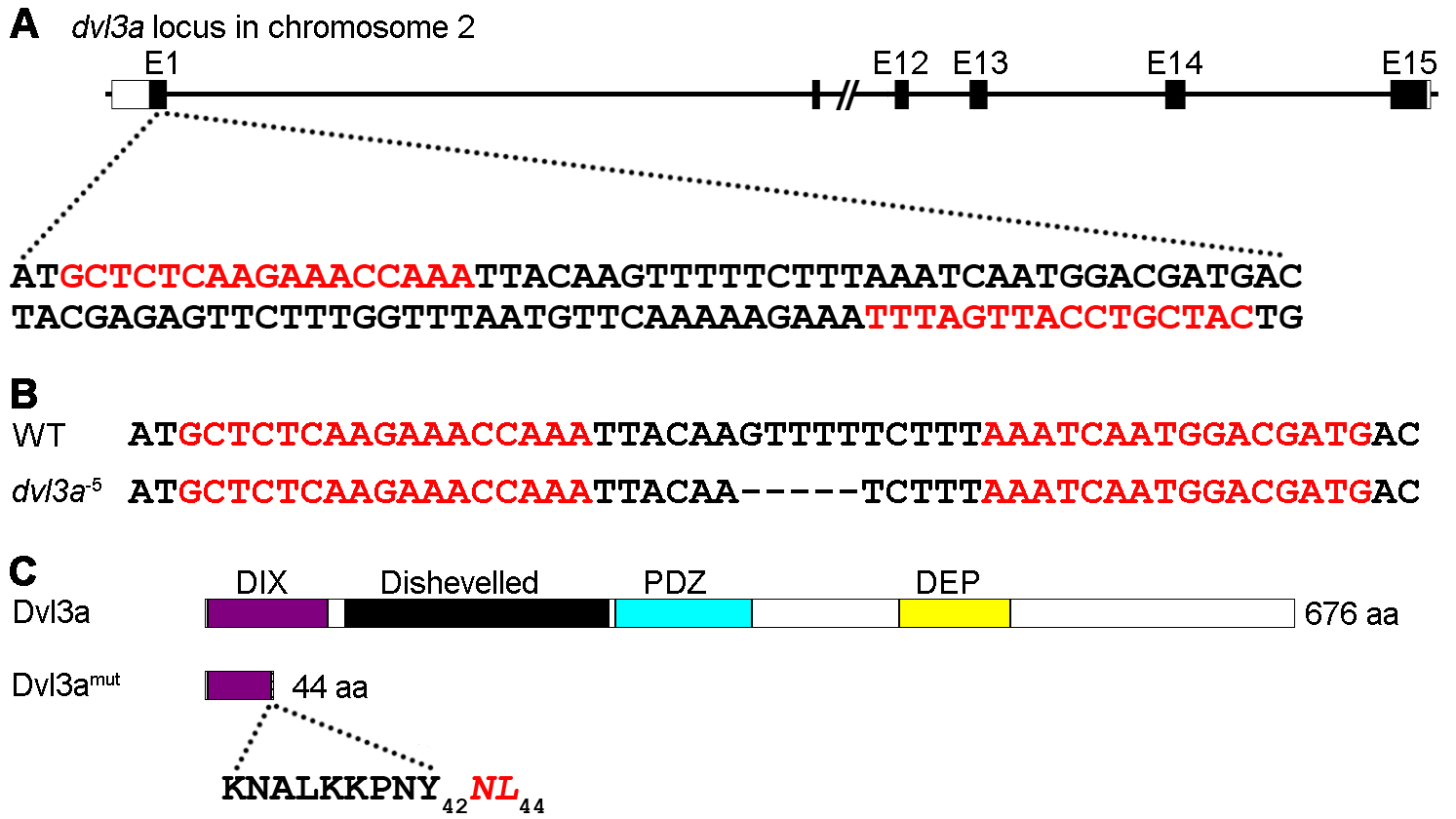

Supplement: S4 Fig — (A) TALENs-targeted sites (red) in the first exon. (B) A deletion of 5 nucleotides (dashes) within the exon. (C) Schematic of Dvl domains shows truncation of Dvl3a protein. (JPG) [file pgen.1007551.s004.jpg]

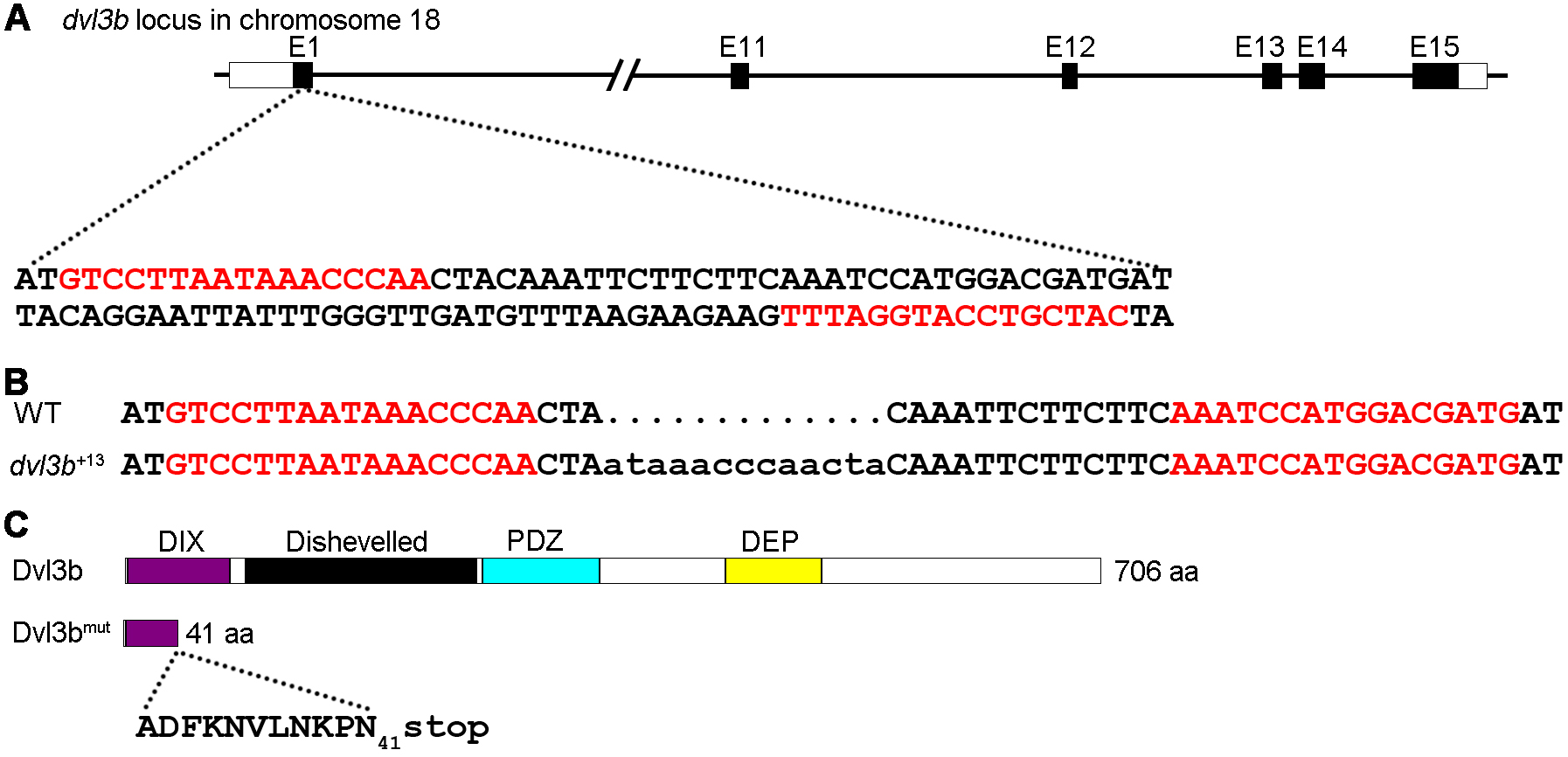

Supplement: S5 Fig — (A) TALENs-targeted sites (red) in the first exon. (B) An insertion of 13 nucleotides (lowercases) within the exon. Dots are introduced in WT sequence to optimize alignment. (C) Schematic of Dvl domains shows truncated Dvl3b protein. (JPG) [file pgen.1007551.s005.jpg]

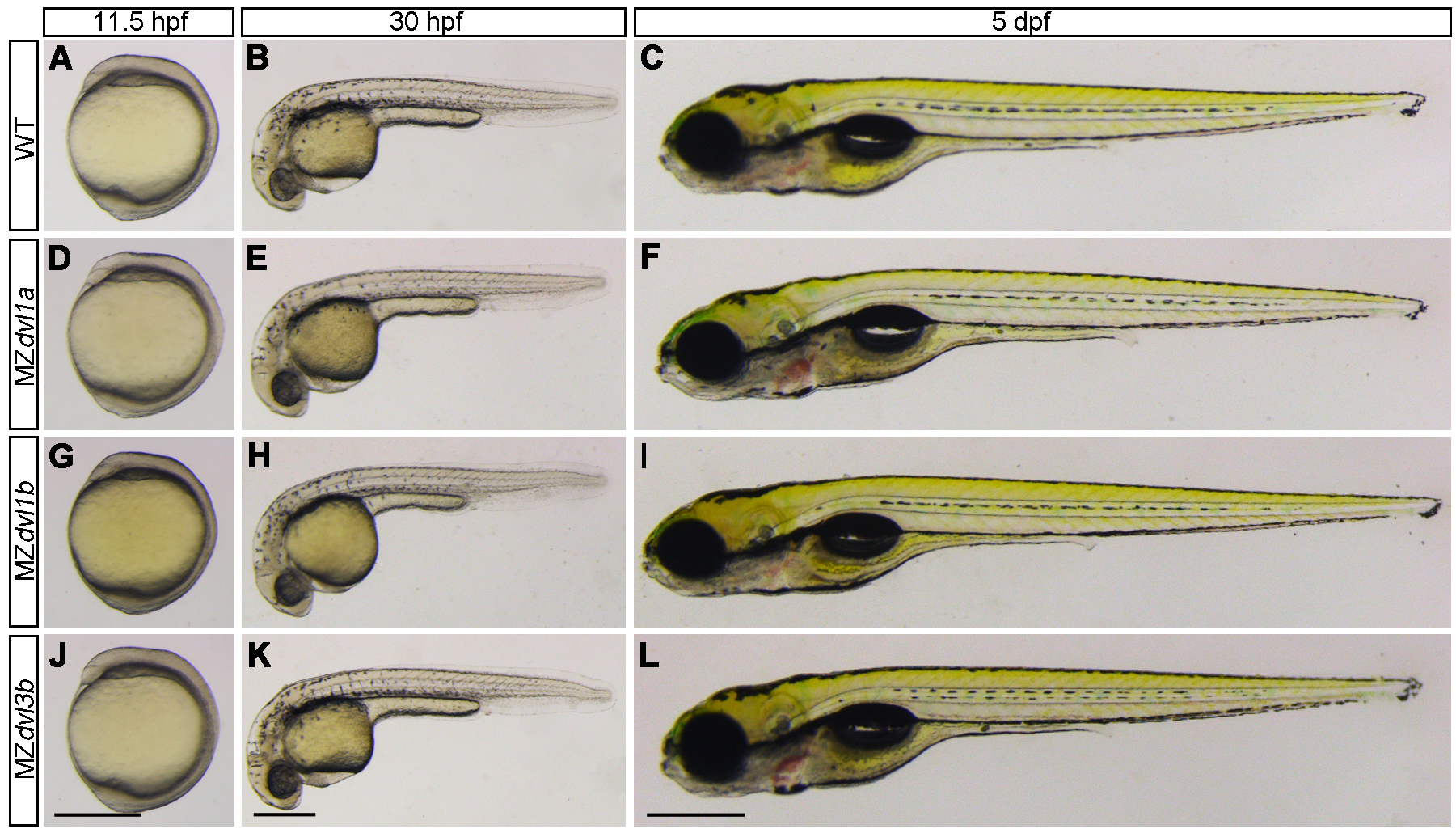

Supplement: S6 Fig — (A-C) WT embryos. (D-F) MZdvl1a mutants. (G-I) MZdvl1b mutants. (J-L) MZdvl3b mutants. All embryos are lateral view. The anterior region of 11.5 hpf embryos is positioned on the top. Scale bars: (A, D, G, J) 400 μm; (B, E, H, K) 400 μm; (C, F, I, L) 400 μm. (JPG) [file pgen.1007551.s006.jpg]

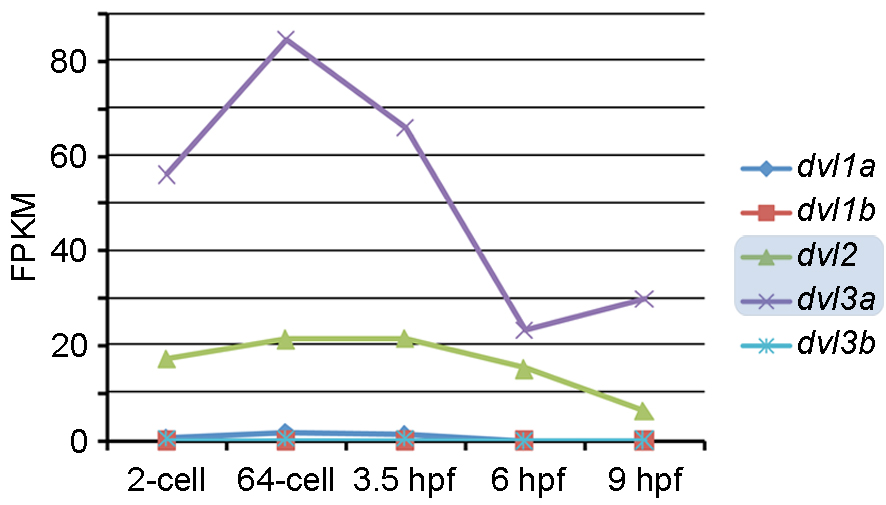

Supplement: S7 Fig — The graph was obtained by analyzing published RNA-seq data (Harvey et al., 2013. See reference 29 in the main text). FPKM, fragments per kilobase million. (JPG) [file pgen.1007551.s007.jpg]

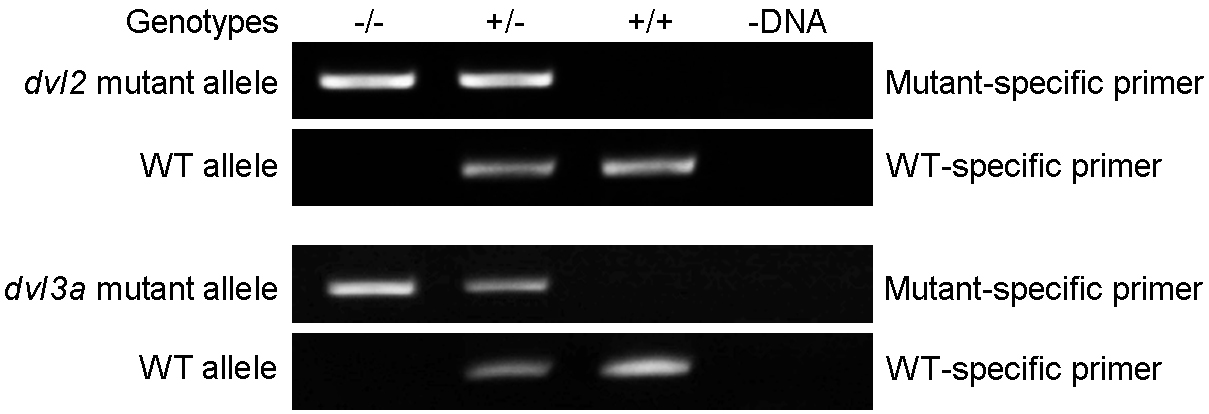

Supplement: S8 Fig — Shown are the PCR products that should be amplified from genomic DNA in WT, heterozygous, and homozygous adult fish, by using allele-specific primers (see S1 Table for primer sequences). (JPG) [file pgen.1007551.s008.jpg]

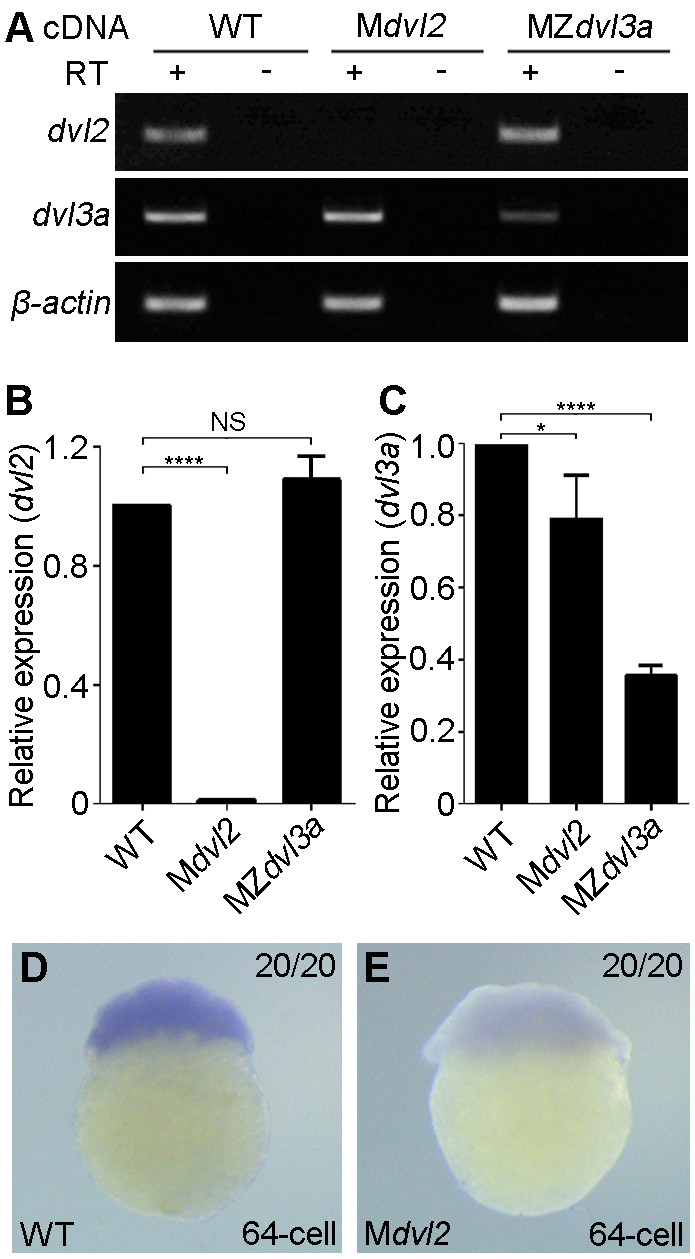

Supplement: S9 Fig — (A) Semi-quantitative RT-PCR analysis to detect mutant dvl2 and dvl3a transcripts at 1-cell stage. ß-actin served as a loading control. NMD can be observed for dvl2 and dvl3a transcripts, respectively. (B, C) Quantification of mutant dvl2 and dvl3a mRNA levels in Mdvl2 and MZdvl3a mutants. The expression level in WT embryo is set as 1 after normalization with ß-actin. Bars represent the mean ± s.d. from three experiments (*, P<0.05; ****, P<0.0001). (D, E) In situ hybridization analysis of dvl2 transcripts in WT and Mdvl2 embryos. RT, reverse transcriptase. (JPG) [file pgen.1007551.s009.jpg]

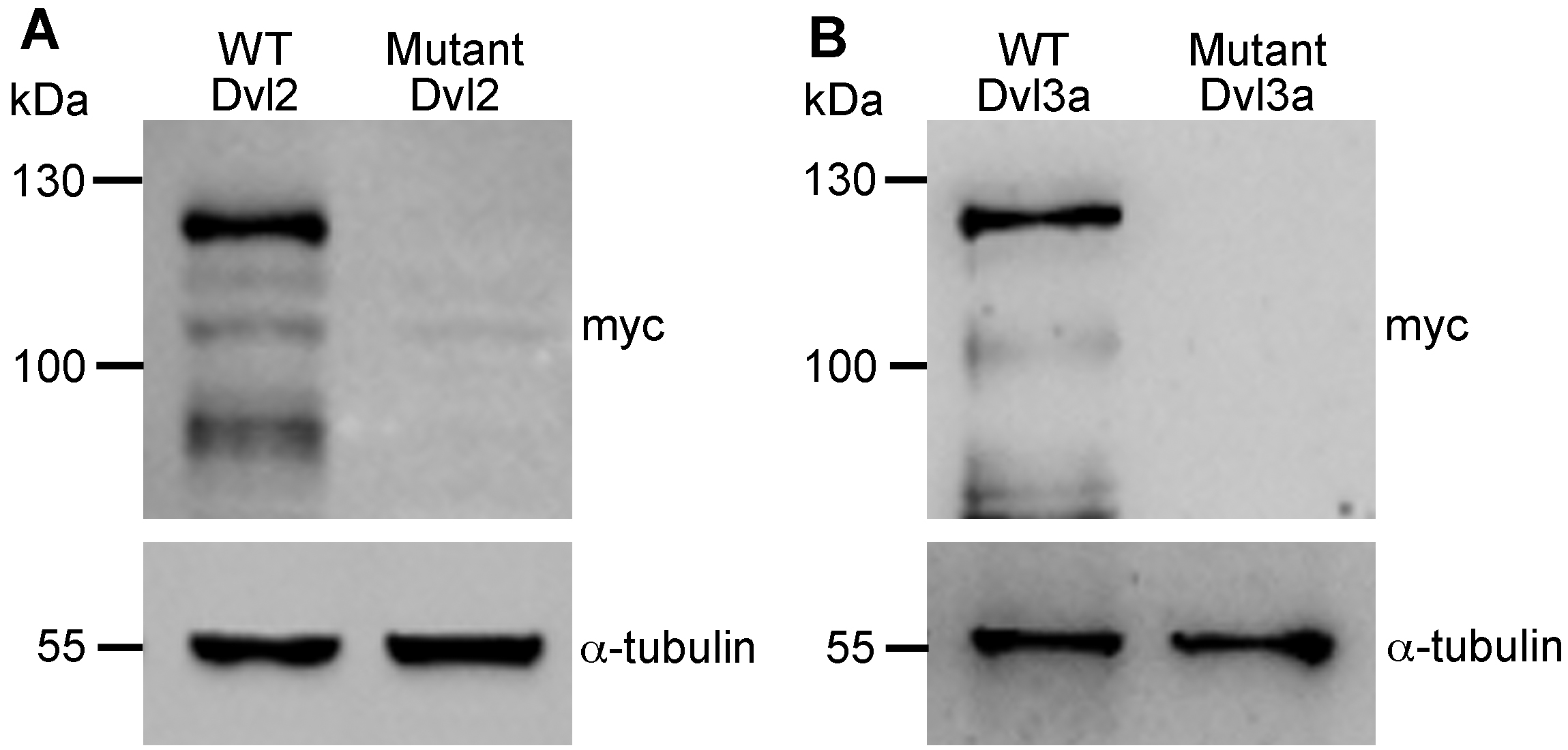

Supplement: S10 Fig — Synthetic mRNAs encoding C-terminally myc-tagged WT and mutant Dvl2 and Dvl3a were expressed in zebrafish embryos. (A, B) Western blotting shows that mutant dvl2 and dvl3a transcripts are not translated. (JPG) [file pgen.1007551.s010.jpg]

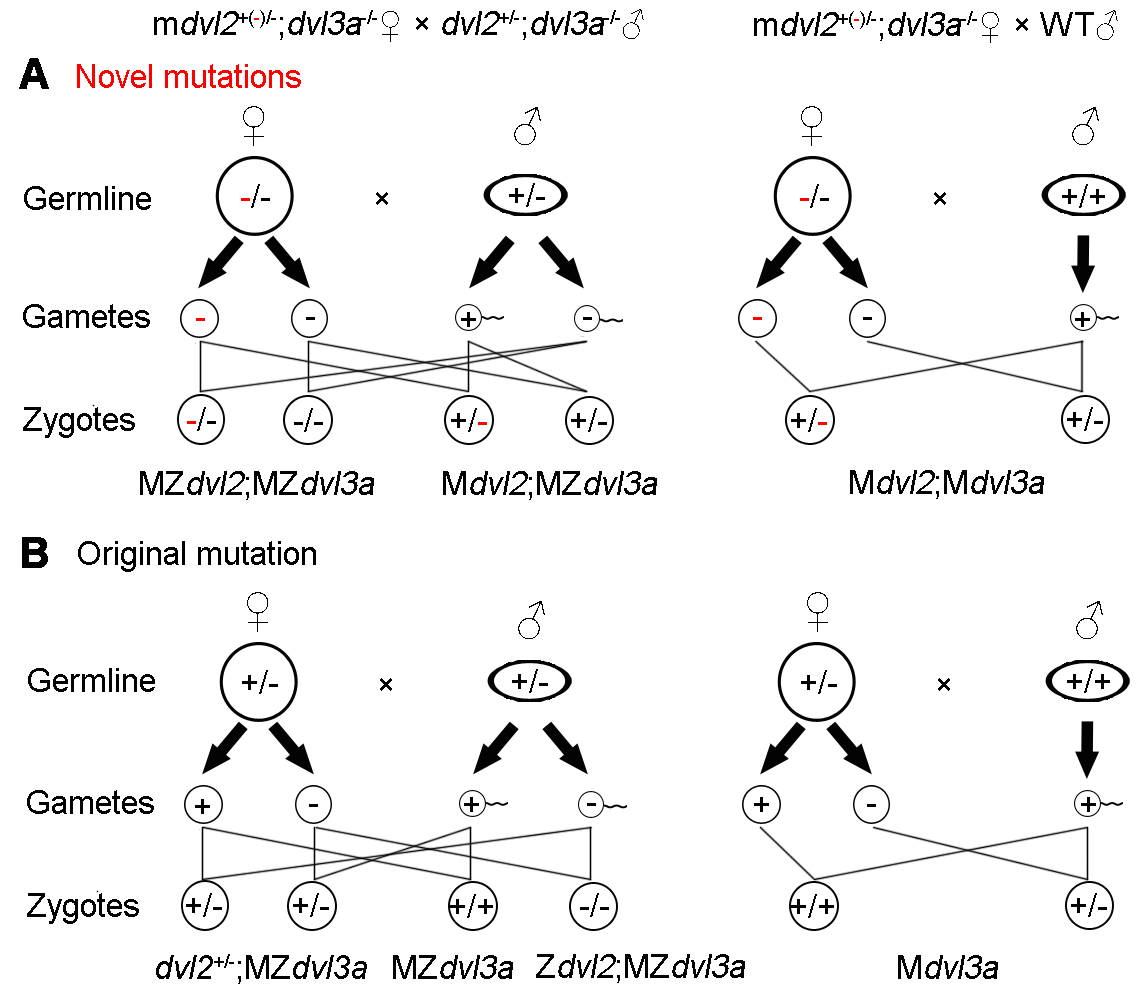

Supplement: S11 Fig — (A) In the crosses between a mosaic female mdvl2+(-)/-;dvl3a-/- fish and a male dvl2+/-;dvl3a-/- fish, when the germline of the female fish contains a novel mutation for dvl2, the gametes that it produces will give rise to an MZdvl2;MZdvl3a offspring when fertilized by a male gamete with the original mutation, and an Mdvl2;MZdvl3a offspring when fertilized by a male gamete with WT dvl2 allele. The same female gametes will give rise to an Mdvl2;Mdvl3a offspring when fertilized by a male gamete from WT fish. (B) When a female mdvl2+(-)/-;dvl3a-/- fish is crossed with a male dvl2+/-;dvl3a-/- fish, if the germline of the female fish only contains the original mutation, the genotypes of offspring should include dvl2+/-;MZdvl3a-/-, MZdvl3a, or Zdvl2;Mdvl3a zygotes, depending on the genotype of the male gamete. These female gametes will give rise to Mdvl3a offspring when fertilized by a male gamete from WT fish. In all these cases, the early embryos should lack half of the dvl2 gene product. Only the dvl2 alleles are indicated in the schema. (JPG) [file pgen.1007551.s011.jpg]

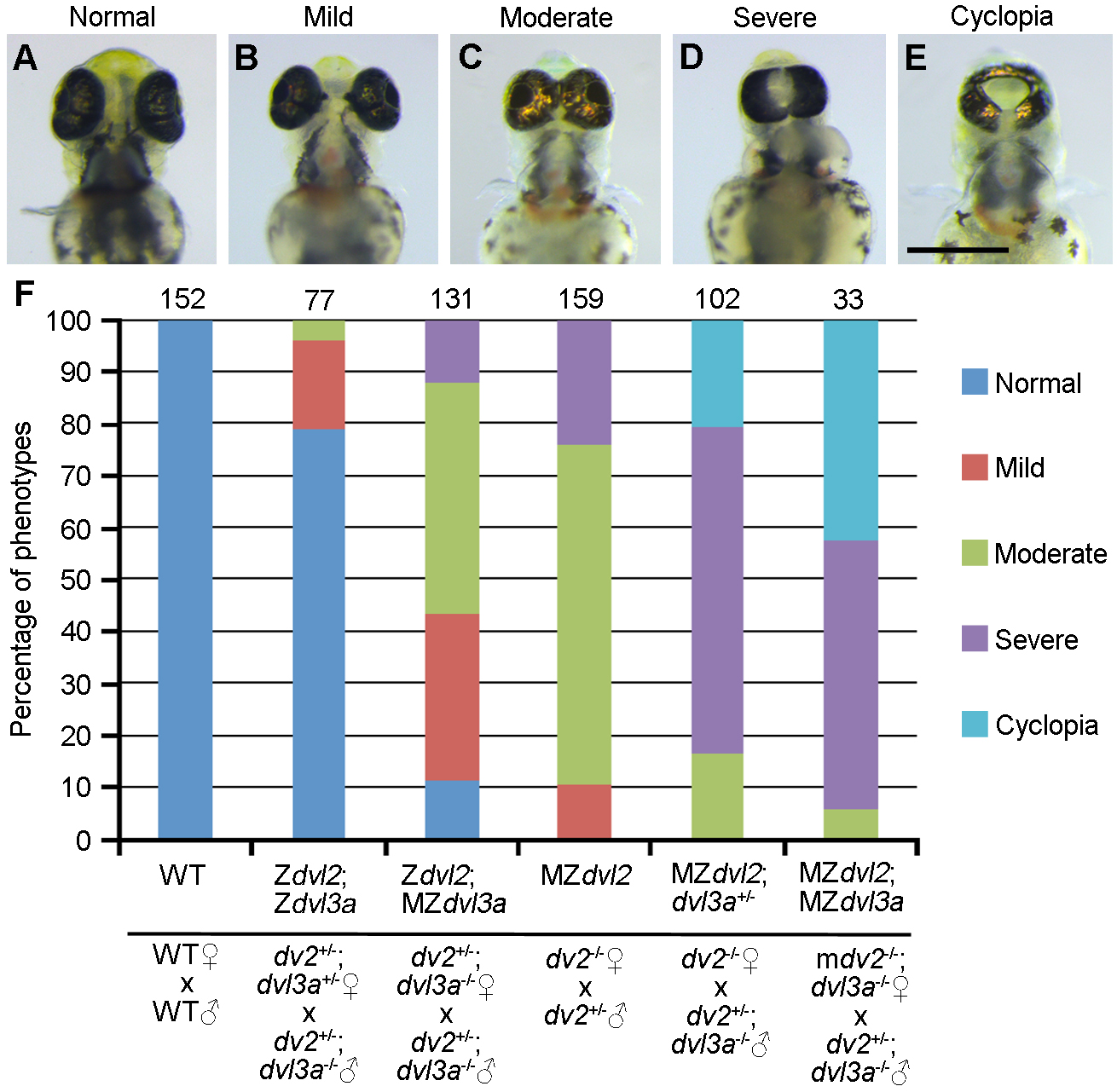

Supplement: S12 Fig — (A-E) Ventral view of representative images of normal and different degrees of eye phenotypes at 3 dpf. (F) Quantitative analysis of different degrees of eye phenotypes in indicated mutants. Except for WT embryos, all mutants were analyzed from three independent crosses using the same fish pair (indicated below the horizontal line). Numbers on the top of each column indicate total embryos carrying the indicated genotypes (above the horizontal line). Scale bar: (A-E) 400 μm. (JPG) [file pgen.1007551.s012.jpg]

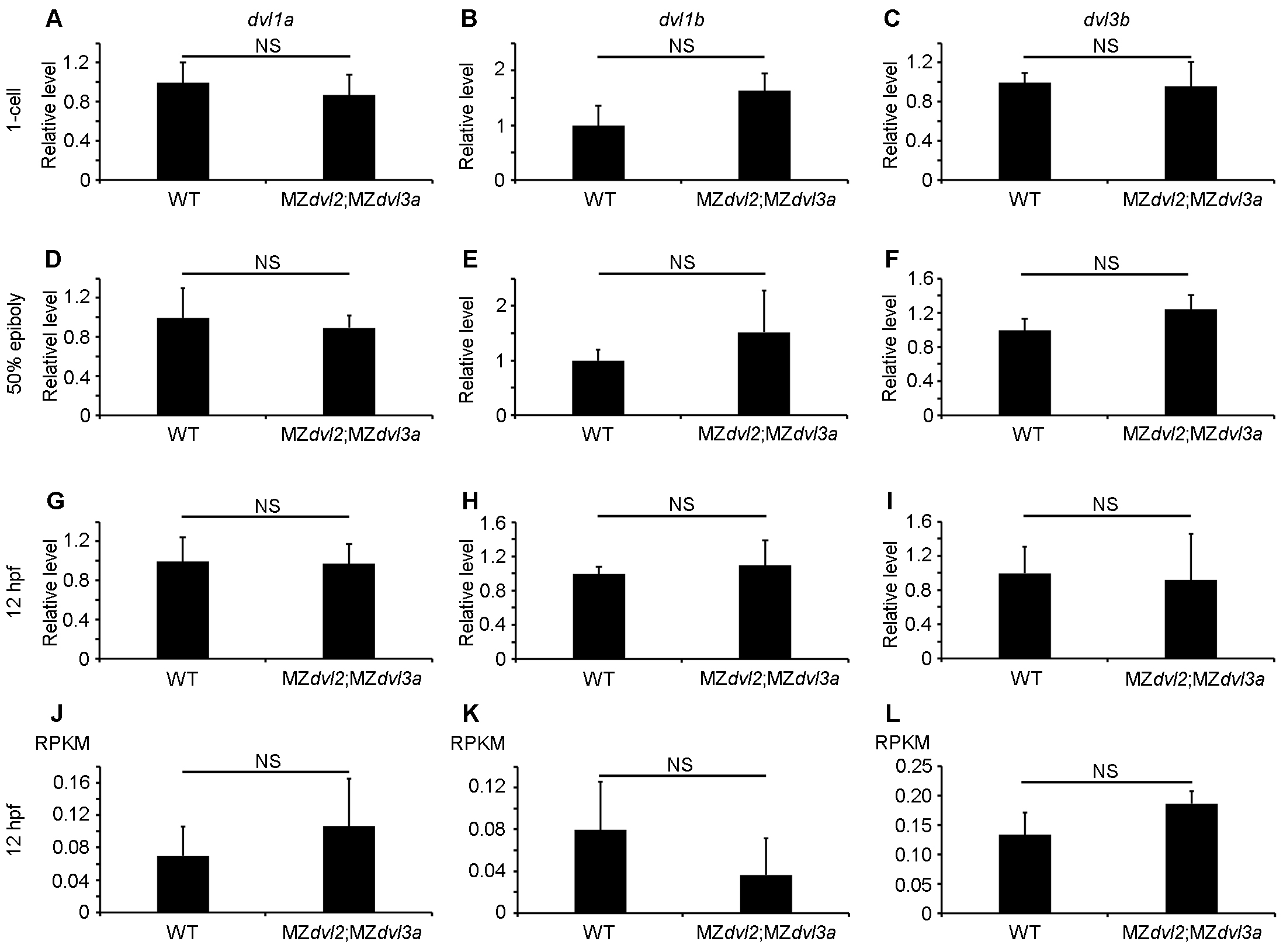

Supplement: S13 Fig — (A-I) Quantitative RT-PCR analysis of dvl1a (A, D, G), dvl1b (B, E, H), and dvl3b (C, F, I) at 1-cell stage (A-C), 50% epiboly (D-F) and 12 hpf (G-I). The expression level in WT embryo is set as 1 after normalization with ß-actin, and bars represent the mean ± s.d. from three independent experiments (NS, not significant). (J-L) Analysis of dvl1a (J), dvl1b (K), and dvl3b (L) expression levels by RNA sequencing at 12 hpf. Bars represent the mean ± s.d. from three independent samples (NS, not significant). RPKM, reads per kilobase million (JPG) [file pgen.1007551.s013.jpg]

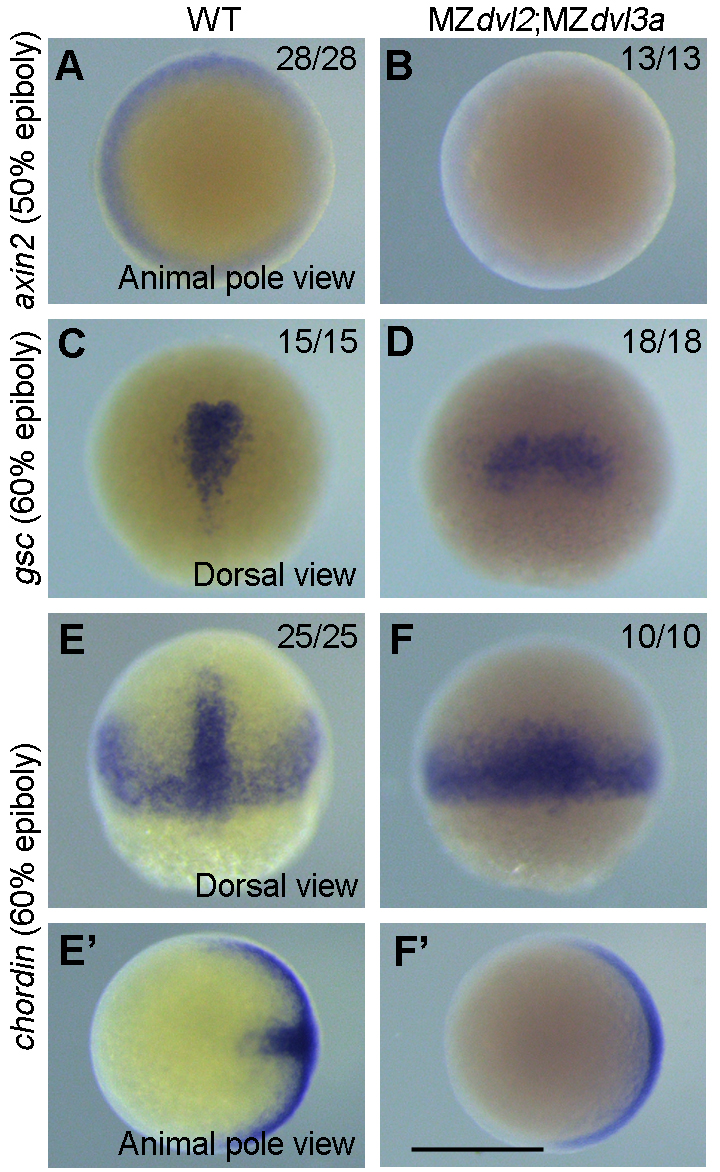

Supplement: S14 Fig — In situ hybridization analysis of dorsoventral markers. (A, B) The expression of axin2 at 50% epiboly is inhibited in MZdvl2;MZdvl3a mutants. (C-F’) The expression domains of goosecoid (gsc) and chordin in MZdvl2;MZdvl3a mutants at 60% epiboly do not show ventral expansion, but reflect impaired AP extension. Scale bar: 400 μm. (JPG) [file pgen.1007551.s014.jpg]

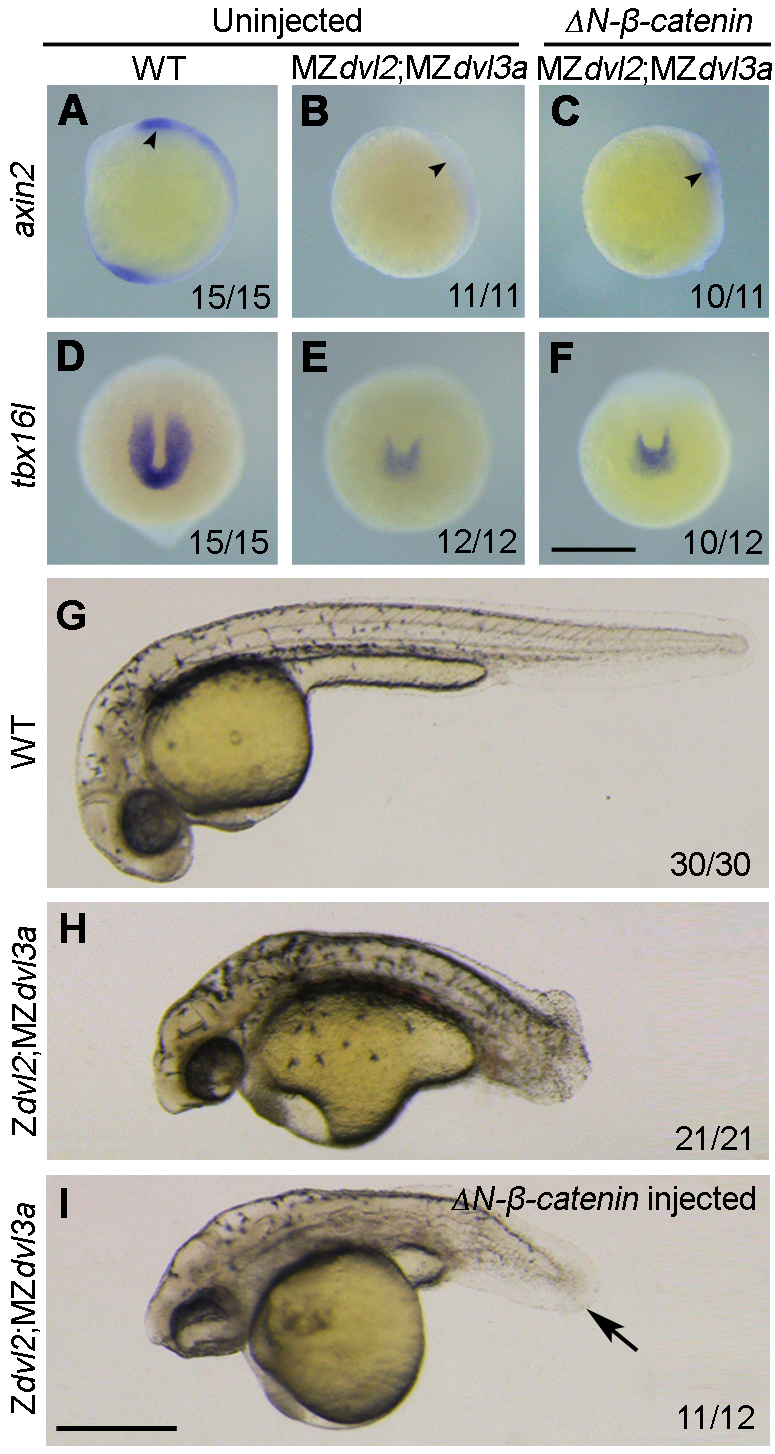

Supplement: S15 Fig — Embryos at 1-cell stage derived from crosses between female mdvl2+(-)/-;dvl3a-/- fish and male dvl2+/-;dvl3a-/- fish were injected with ΔN-ß-catenin mRNA (50 pg). Following in situ hybridization or phenotype analysis, the embryos were subjected to genotyping. (A-F) ΔN-ß-catenin partially rescues axin2 and tbx16l expression in MZdvl2;MZdvl3a mutants. Arrowheads indicate the axin2 anterior expression domain. (G-I) ΔN-ß-catenin partially rescues tail development in Zdvl2;MZdvl3a mutants (arrow). Scale bar: (A-F) 400 μm; (G-I) 400 μm. (JPG) [file pgen.1007551.s015.jpg]

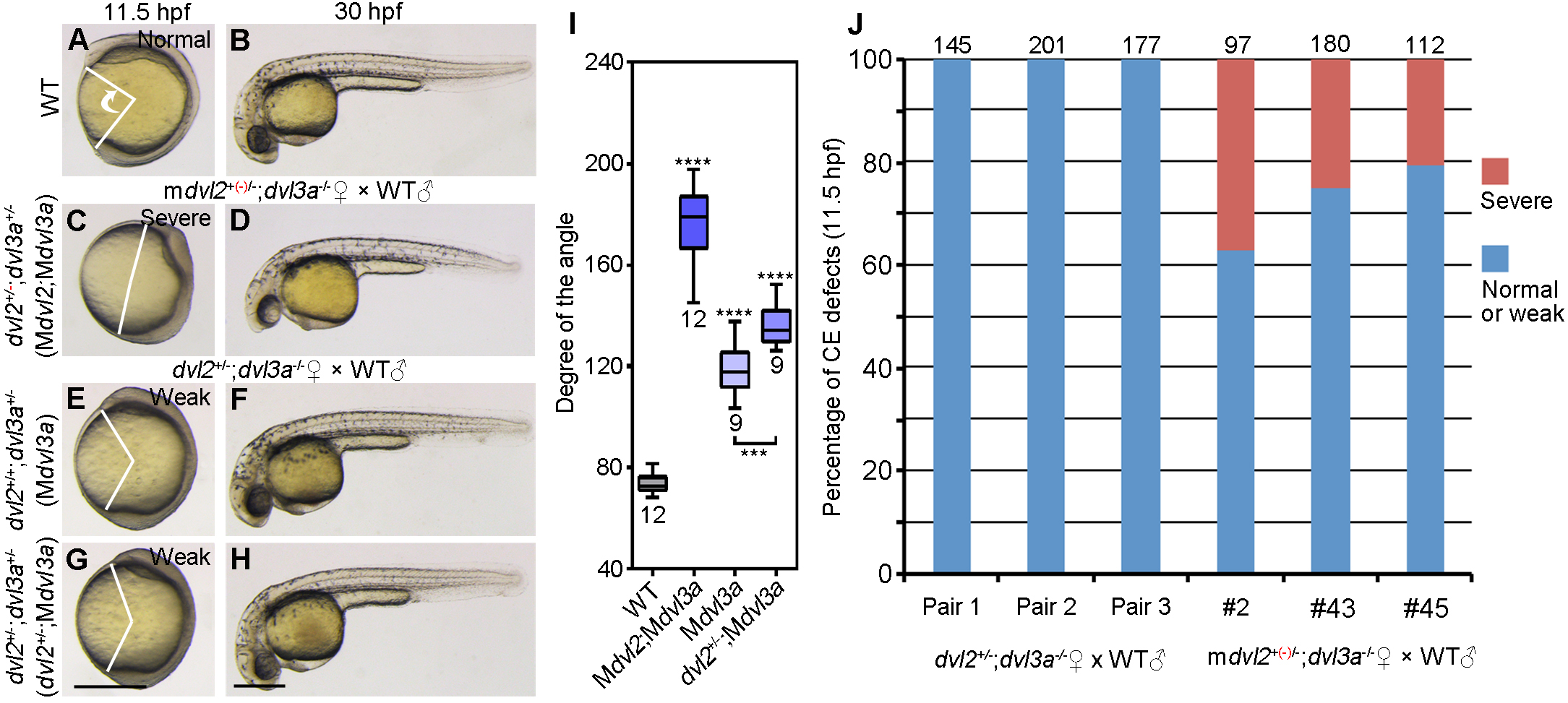

Supplement: S16 Fig — The embryos were imaged at 11.5 hpf and 30 hpf, followed by genotyping, and the extent of axis extension defect was reflected by the angle between the anterior end and the posterior end at 11.5 hpf. (A, B) WT embryos. (C, D) Mdvl2;Mdvl3a mutants from crosses between female mdvl2+(-)/-;dvl3a-/- fish and male WT fish were genotyped for the presence of a novel indel (red) along with a WT allele in the dvl2 locus. They have dvl2 and dvl3a heterozygous mutations. (E, H) The offspring with the two possible genotypes (dvl2+/+;dvl3a+/- and dvl2+/-;dvl3a+/-; the effects of these mutations are indicated in parenthesis), derived from a cross between female dvl2+/-;dvl3a-/- fish and male WT fish, are maternal mutants for dvl3a, with a reduced dosage of maternal dvl2 in both cases, despite of the genotype. (I) Statistical analysis of the extent of axis extension delay in three types of maternal mutants. Bars represent the mean ± s.d. from indicated numbers of embryos, and asterisks above the bars show significance with respect to WT embryos (***, P<0.001; ****, P<0.0001). (J) Quantitative analysis of defective axis extension at 11.5 hpf. Each type of cross was done using three independent fish pairs, and total numbers of embryos analyzed are indicated on the top of each column. Subjective measures of axis extension defect are shown on the embryos at 11.5 hpf. Scale bar: (A, C, E, G) 400 μm; (B, D, F, H) 400 μm. (JPG) [file pgen.1007551.s016.jpg]
