# Supplementary material for: Mutational analysis of dishevelled genes in zebrafish reveals distinct functions in embryonic patterning and gastrulation cell movements
Source: PLoS Genet. 2018 Aug 6;14(8):e1007551. doi: 10.1371/journal.pgen.1007551 (PMC6095615; doi:10.1371/journal.pgen.1007551)
Supplement: S1 Table — (DOCX) [file pgen.1007551.s018.docx]

**S1 Table**. Primers for semi-quantitative PCR, quantitative PCR and allele-specific PCR

| Primer names | Sequences | Purposes |
| --- | --- | --- |
| *dvl1a*-F  *dvl1a*-R | 5′-GGGTTCAAAAATCTGTGGAG-3′  5′-GTGAAGCGATGTGAATACTTT-3′ | Genomic and allele-specific PCR (Difference in size between WT and mutant allele) |
| *dvl1b*-F1  *dvl1b*-F2  *dvl1b*-R | 5′-CTTTCGGATATGACACTGCC-3′  5′-AACACAGGCAGCAAAACCTC-3′  5′-GCCATCAATGACAGCTTCG-3′ | Genomic PCR |
| *dvl2*-F1  *dvl2*-F2  *dvl2*-R | 5′-CGACATTGTCCAAGAGAGC-3′  5′-TTGTGGTCTAGACGACTTTCC-3′  5′-TGCGATGATGGCTTGATACG-3′ | Genomic PCR |
| *dvl3a*-F1  *dvl3a*-F2  *dvl3a*-R | 5′-TACGGTTGATAGCCATCCAG-3′  5′-AAGATTCCTGGGAACTCCG-3′  5′-GGACCAAACATTCACCCCTG-3′ | Genomic PCR |
| *dvl3*-F1  *dvl3*-F2  *dvl3*-R | 5′-CGAAATGAATCCCATCCA-3′  5′-ACGTACTTACGACAGCAAGG-3′  5′-GGGTTTCCATGTAAAACGC-3′ | Genomic PCR |
| *dvl1b*(mut)-F  *dvl1b*(wt)-F | 5′-CAACCGACCGGTATAAATTC-3′  5′-GCAACCGACCGGTCAACAGC-3′ | Allele-specific PCR (Used with *dvl1b*-R) |
| *dvl2*(mut)-R  *dvl2*(wt)-R | 5′-CACAATCACTTACCCCCTGG-3′  5′-CAATCACTTACCCGAAGTCC-3′ | Allele-specific PCR (Used with *dvl2*-F1, *dvl2*-F2) |
| *dvl3a*(mut)-F  *dvl3a*(wt)-F | 5′-CAAGAAACCAAATTACAATC-3′  5′-AACCAAATTACAAGTTTTTC-3′ | Allele-specific PCR (Used with *dvl3a*-F1, *dvl3a*-F2) |
| *dvl3*(mut)-F  *dvl3*(wt)-F | 5′-CCTTAATAAACCCAACTAAT-3′  5′-TTAATAAACCCAACTACAAA-3′ | Allele-specific PCR (Used with *dvl3*-R) |
| *dvl2*-F  *dvl2*-R | 5′-ATTGGAGACTCCAGACCTCCT-3′  5′-AGCTACAGCTCCTCCCTTCAT-3′ | RT-PCR |
| *dvl3a*-F  *dvl3a*-R | 5′-CTCCGATCTCAACAAGCGAG-3′  5′-AACATCCATCTGAGTGGGAGC-3′ | RT-PCR |
| *dvl1a*-F  *dvl1a*-R | 5′-ACATGGCTTCTCTCAATCTG-3′  5′-TGTGAAAACTGTGGCAGGGA-3′ | qPCR |
| *dvl1b*-F  *dvl1b*-R | 5′-CTGTTCTCCGGCAATTGTTC-3′  5′-GGTTGCTGAGGACATTTTTG-3′ | qPCR |
| *dvl3*-F  *dvl3*-R | 5′-AGTCGAAGCAGCGGCTCTAA-3′  5′-ATGCTGCGGATGGTGTGTGA-3′ | qPCR |
| *ß-actin*-F  *ß-actin*-R | 5′-CACAGTGCTGTCTGGAGGTAC-3′  5′-GAGGGCAAAGTGGTAAACG-3′ | RT-PCR and qPCR |
